# Predicting standardized absolute returns using rolling-sample textual modelling

**DOI:** 10.1371/journal.pone.0260132

**Published:** 2021-12-07

**Authors:** Ka Kit Tang, Ka Ching Li, Mike K. P. So

**Affiliations:** Department of Information Systems, Business Statistics and Operations Management, The Hong Kong University of Science and Technology, Hong Kong, Hong Kong; University of Delaware, UNITED STATES

## Abstract

Understanding how textual information impacts financial market volatility has been one of the growing topics in financial econometric research. In this paper, we aim to examine the relationship between the volatility measure that is extracted from GARCH modelling and textual news information both publicly available and from subscription, and the performances of the two datasets are compared. We utilize a latent Dirichlet allocation method to capture the dynamic features of the textual data overtime by summarizing their statistical outputs, such as topic distributions in documents and word distributions in topics. In addition, we transform various measures representing the popularity and diversity of topics to form predictors for a rolling regression model to assess the usefulness of textual information. The proposed method captures the statistical properties of textual information over different time periods and its performance is evaluated in an out-of-sample analysis. Our results show that the topic measures are more useful for predicting our volatility proxy, the unexplained variance from the GARCH model than the simple moving average. The finding indicates that our method is helpful in extracting significant textual information to improve the prediction of stock market volatility.

## Introduction

Risk management has always been important for financial institutions and individual investors. Globalization has led to growing interconnections between markets in terms of their economies, financial markets and political environments; the global coronavirus outbreak in 2020, for example, has put a halt to operations in many countries and is potentially one of the major reasons behind stock market crashes in March, 2020. Meanwhile, it is noticeable that online searches for coronavirus have been increasing and had even appeared before the outbreaks. Therefore, we believe it is worthwhile to investigate whether textual information can provide some hints on predicting risks and facilitating risk management.

Textual information can be accessed from different sources such as annual reports, financial news, and even on the search engines such as Google. For example, [1] showed that changes in online behaviour relating to politics and business on Google and Wikipedia were historically linked to stock market moves. [2] used Twitter data to predict public mood and used that data with the Dow Jones Industrial Average (DJIA) values to predict the stock movements. [3] captured news through sentiment, entropy and the topical context to forecast volatility and drawdown risk. [4] showed that the topics extracted from news can predict trading activity by using a simple regularized regression. In addition, many studies have proven the usefulness of textual data in terms of predicting market volatility. For example, studies such as [5–7] have shown that textual data enhances the volatility prediction models.

In general, as suggested by [8], the two main approaches to processing textual data are lexicon-based, such as the bag of words using the Loughran and McDonald dictionary [6] and machine learning algorithms. For instance, [9] extracted news sentiment to predict stock prices using long short-term memory. [10] quantified the semantic information in news about a company by using latent semantic analysis to predict volatility. In addition, there are many studies based on using bag-of-words or sentiment analysis to extract the information from textual data. However, we believe that besides conveying the meaning by each word, or by possible positive and negative sentiments, news should also convey certain semantic information to the readers, (e.g., topics). Thus, this research focuses on whether the context of news can help to predict market volatility.

Predicting volatility is the key to risk management as it helps investors to adjust their portfolios [11] and trading strategies [12], and therefore researchers have introduced many tools in volatility predictions. Years ago, the well-known Generalized Autoregressive Conditional Heteroskedasticity (GARCH) models captured and predicted the volatility and performance of stocks using their historical prices. However, with the development of machine learning, people can now better utilize further information to gain market insights. For example, [13] summarized information from the price of components and macro-economic indices in only one model to achieve notable market index prediction. Therefore, we are particularly interested in using other information including all kinds of news related to the market such as political, environmental, social and financial events in predicting volatility, By making use of statistical methods and machine learning models such as structural equation modelling [14] and LDA, we can gain more understanding of the textual information.

LDA is a statistical topic model proposed in [15], able to analyze hidden topics in large scale data. For example, [16] utilized LDA to extract topics from Twitter concerning Italian banks and to provide insights on each topic using visualization such as word clouds to convey the information. In addition, because of the dynamic features in textual data overtime, there are many variations of LDA that have incorporated the time series concept. For example, [17] analyzed the time evolution of topics at a discrete time interval of one year, and [18] focused on topic detection and tracking on conversational content using conceptual dynamic LDA. Some studies have focused on how to scale up the use of dynamic topic modelling. For instance, in order to enable the dynamic topic modelling to be more applicable to a larger dataset, [19] made use of the Stochastic Gradient Langevin Dynamics and Metropolis-Hastings sampler to enhance the model performance. [20, 21] constructed the Clustered Latent Dirichlet Allocation (CLDA) to take advantage of parallel resources that not only increase the speed of computation and facilitate the use of large corpora, but also allow the number of topics in each discrete time period to change. [22] used different kernels to enhance to the interpretability of the model results and increase its usefulness in events detection. All these dynamic topic models required a large dataset for statistical learning and sophisticated statistical computation techniques for analyzing the dynamic topics models as the time evolution feature of topics is explicitly integrated in the statistical models to understand possible structures in topic evolution from all textual data in the sample.

In this paper, we utilize the existing resources of a latent Dirichlet allocation model proposed by [15] with a rolling window extension that we have called rolling LDA and apply a concept similar to [17] but with a smaller discrete time interval to capture the dynamic features in textual data. Unlike the dynamic topic model in [19–21], using a rolling window approach allows us to use a textual dataset of a moderate size because we only need the most updated textual data to build the LDA. For example, the window size adopted is 30 days with a rolling size of 1 day. By capturing the news data of one day, we can identify the semantic features of the news at time *t* based on updated news data.

Different topic models have been used for market prediction;, for example, [23] showed that using LDA, financial news can better predict the direction of volatility in the U.S. market than stock prices, and [24] created sentiment scores from LDA to explain stock return and trading volume. However, to the best of our knowledge, no dynamic topic model has been used to predict market volatility with the exceptions of [25, 26]. The former made use of the multiscale dynamic topic model developed by [27] to define a topic score that is incorporated into time series models of volatility as an exogenous variable to enhance the model. The latter developed a financial LDA to incorporate changes in time series and extract useful features from topic distributions for prediction using back propagation neural network and support vector regression. As there is no extensive research on further utilising the statistical properties from LDA for prediction purposes especially in financial markets, we aim to investigate whether the results from LDA such as topic distributions and word compositions can provide extra explanatory power to the volatility in the financial market.

Due to the fluctuating volatility of financial markets [28], a non-linear model should be adopted as well [29]. Thus, we also adopt a rolling window scheme in a regression model to predict the market volatility proxy which is the log of absolute standardized GARCH residuals. In this study, we use the Hang Seng Index (HSI) of Hong Kong as an example to capture the overall market behaviour in Hong Kong.

The literature commonly defines the latent and potential topics to which a document belongs. Examples include [30, 31], in which the authors tend to investigate what each topic represents by looking at their word composition of the topics and how these topics infer the findings. However, the latent topics become subjective with human interpretations, not to mention the fact that it requires a lot of human resources to interpret the result from a rolling window model. Furthermore, as topics evolve and change and the word compositions in the same topic can be different in different periods, it can be difficult for humans to link the topics from different periods. Therefore, we also introduce a more reliable and systematic method to utilize the statistical properties of the topics and the word distributions generated from the rolling LDA model to summarize and learn the evolution of the topic through our proposed topic score measures. As the constructed topic score measures can be regarded as predictors for regression, we can further utilize the results from LDA and perform regressions to predict the market volatility.

In summary, in this paper we present the development of a machine learning pipeline for market volatility prediction, making use of topic modelling and a simple regression model on the news collected. In the prediction model, we first introduce a methodology utilizing the distribution outputs from an LDA with a rolling window extension and develop ways to summarize the textual information in a systematic manner over time rather than relying on the human interpretation usually found in the literature. We then develop different decision rules to enhance the market volatility prediction based on a rolling window regression model. In the empirical study, we predict the volatility of the Hong Kong stock market by acquiring textual data from open news sources, as well as the sources obtained from a licensed database of Reuters, and the closing prices of the HSI. The positive results reported in this papers demonstrate firstly, the usefulness of the model using textual information to predict volatility by comparing it to a benchmark model, and secondly, the usefulness of the semantic information capturing process based on the statistical properties of the output generated from the rolling LDA model.

## Methodology

**Fig 1:**
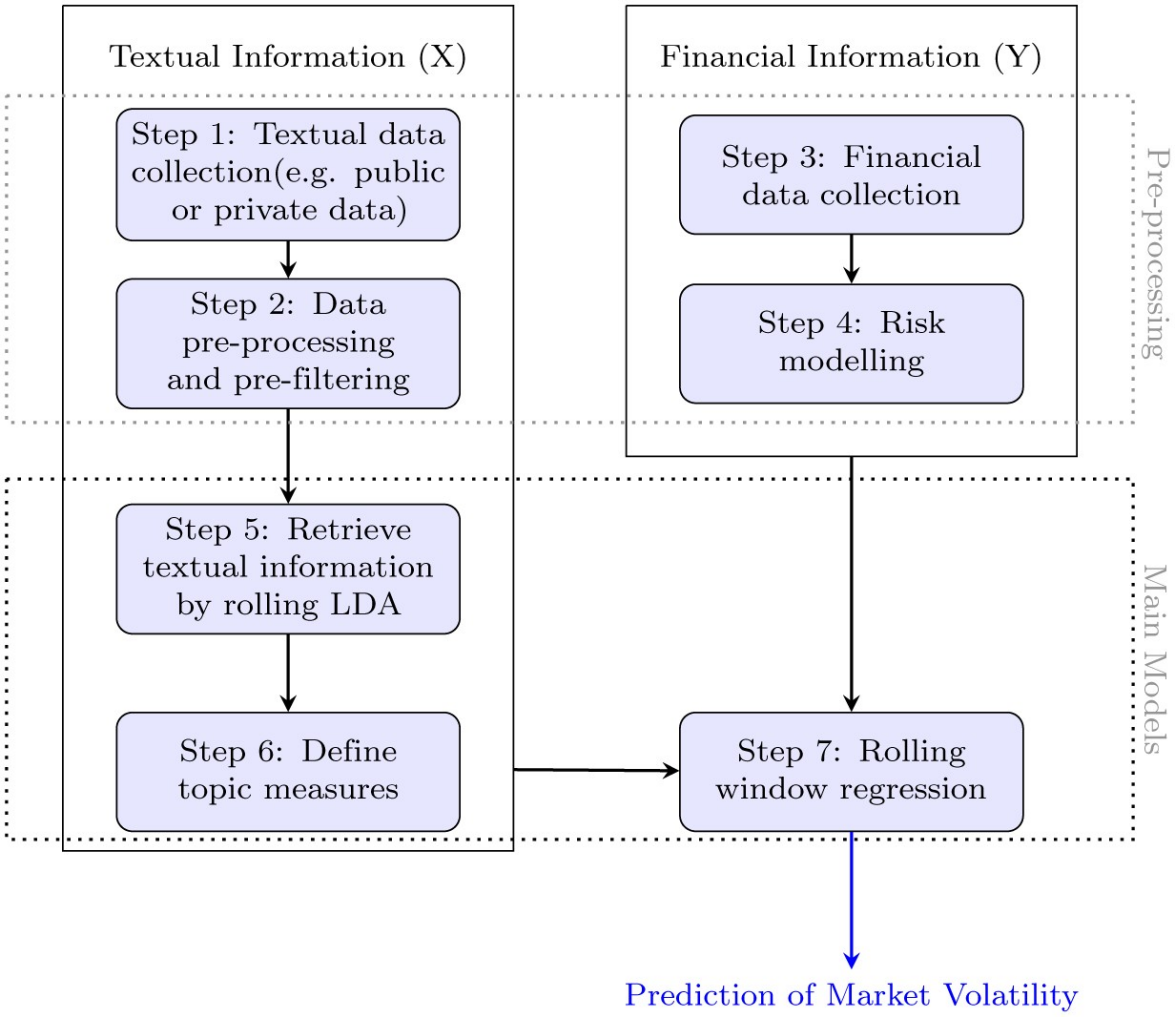
Methodology flow chart. An overview of the entire research methodology from data collection to market volatility prediction. The methodology consists of seven steps, with the aim of making a prediction on market volatility.

This section aims to examine the relations between risk in the financial markets and textual news information and whether the unstructured textual information can provide a better prediction of the market volatility. An overview of the methodology flow we adopted in this study is shown in Fig 1. Two types of data—financial data and textual data are collected. The collected data should also undergo some data cleansing processes to ensure the quality of the model input. The entire pre-processing process is unique and customized for different data inputs, as indicated by the “pre-processing” block shown in Fig 1. The cleaned textual data will undergo a feature engineering process using the rolling latent Dirichlet allocation and a statistical approach to define the topics before acting as predictors. These predictors help to predict the response, the market volatility which is an information extracted from the financial data, using a rolling regression model. A more detailed explanation of the methodology is presented below.

The data collection can be subjective in our methodology(Fig 1: Step 1). The progress of the data collection process is highly flexible—different kinds of corpus (e.g. news, articles, messages) can be treated as raw input in this machine learning pipeline. In our empirical study, we scrape economic related news from public websites for the experiment, although, theoretically, all kinds of textual data can be fitted but may yield different results. Similarly, the data cleansing process (Fig 1: Step 2), particularly in the textual data, should typically be customized for the collected data in order to achieve better model performance. The approach to the data cleansing process used in this study is discussed in the Empirical study section. When both the textual and financial data have been cleaned, they can be further processed to become meaningful predictors and responses, respectively. In the case of forming the response (Fig 1: Step 3 and Step 4), of the market volatility, such a measure can be withdrawn from the financial data through different processes. However, in the case of forming the predictors, this study takes a different approach from the existing papers to extract information from texts as we want to capture the dynamic features of the textual data in the corpus. Examples of the collected textual data are presented in Table 1 showing the collection of news related to Hong Kong and from around the globe. The news categories, including economy, political and health, to name but a few, seem to be very different. For example, some content is consistent over all periods, such as the performance of stocks or indexes in Hong Kong, China or the APAC region, whereas some topics, such as trade war, Brexit or coronavirus only present in certain periods. For this reason, we mainly utilize the changing statistical output from latent topics in the textual data which is laid out in details in Step 5 and Step 6 in Fig 1. Hence, in the Methodology section, we will focus on Step 5, Step 6, and Step 7 in Fig 1 to accentuate what we have done in summarizing the information from the textual data and using it to predict market volatility.

**Table 1:** Examples of news information collected from open sources.

Date	Title	Content
4/5/2016	TREASURIES-U.S. Treasury yields fall as weak growth data bolsters bonds	Longer-dated U.S. Treasuries fall to one-month lows * Weak U.S. trade data, EU PMI spur fears of growth slowdown * Continued Brexit fears add to global uncertainty … Pond added, noting that fears Britain could leave the European Union in a June 23 vote remained a cause of uncertainty. “The June referendum is keeping markets on edge and that is likely to keep a bid to safe-haven assets for a bit here.” Chicago Fed President Charles Evans also talked about the worrying possibility of a so-called “Brexit” vote in Britain as well as about the “large uncertainty” being generated by the ongoing U.S. presidential election. “It’s hard to know what risks might be hitting us…
7/19/2016	China state banks sharply push up yuan after key level breached	“The central bank turned into attack mode in the afternoon,” said a trader at a Chinese commercial bank in Shanghai. “It might be angry about all the publicity around yuan depreciation.” The yuan’s brief slide past the 6.7 milestone came on the eve of the U.S. Republican National Convention, where presumptive presidential nominee Donald Trump is to be formally announced. In June, Trump called China a “grand master” of currency devaluations and urged a tax on imports. The moves also come just days before China hosts G20 finance ministers and central bank governors, who in April reaffirmed a pledge to not set exchange rates for competitive purposes ‥
6/28/2017	Hong Kong police arrest democracy activists ahead of Xi Jinping visit	Hong Kong police have arrested protesters on the eve of Chinese President’s visit to the city to commemorate the 20th anniversary of its handover from Britain to China.”, “The demonstrators had gathered around the Golden Bauhinia statue to express their frustration with what they perceive as Beijing’s encroachment on democratic values…
3/5/2018	Tech giants challenge HKEX reform provisions	The listing proposal is seen clashing with draft rules in the Mainland.’,’Bloomberg reports that technology companies and service providers are reportedly questioning key areas of Hong Kong Exchanges and Clearing Ltd.’s plan to allow dual-class shares.’,’The regulator is mulling revisions to its rules so that company founders can remain in control after listing but this may pose problems for China’s tech titans who employ a variable interest entity (VIE) structure like Xiaomi and Tencent Music Entertainment…
4/4/2018	China stocks up on hopes full-blown trade war with US can be averted; HK flat	China and Hong Kong stocks shook off the Trump administration’s tariff announcement against Chinese exports, as investors judged the widely-expected move would have negligible impact on growth, and that a full-blown trade war will be averted through negotiations. Late on Tuesday, the Trump administration announced 25 percent tariffs on $50 billion of annual imports from China, covering around 1,300 industrial technology, transport and medical products…
8/31/2018	HK stocks fall on renewed trade war fears; Tencent slumps	Aug 31 (Reuters)—Hong Kong stocks ended lower on Friday, falling for four months in a row, with risk appetite curbed by renewed trade war fears, while the China Enterprises Index lost 0.8 percent, to 10,875.58 points.’,’** Sentiment was hit by a report that U.S. President Donald Trump is prepared to ramp up a trade war with China and is ready to impose tariffs on $200 billion more in Chinese imports after a period of public comment on the plan ends next week…
3/21/2020	China’s imported coronavirus cases soar as students, expats flock home	SHANGHAI/BEIJING (Reuters)—China reported a record rise in imported coronavirus cases on Friday as students and expatriates returned home from the United States and Europe, sparking fears of a second wave of infections just as the country recovers from the initial outbreak…

This table shows some examples of the textual information collected from different open sources data with a focus on search keywords related to Hong Kong and indicates that there is a wide range of topics over time. For instance, news related to trade war, Brexit, U.S. Presidential Election, Hong Kong democracy and coronavirus were some of the popular topics at some point over the five years period of news collection.

### Retrieve textual information by rolling LDA (Fig 1: Step 5)

Before discussing how the rolling LDA works in this study, the matrix below shows what the input of the model should look like after the data cleansing procedures conducted in Step 2. The input is basically a document term matrix (DTM) with a dimension of *M* x *N*, which is denoted as *D* here:

$$D=[\begin{array}{ccc} {\tilde{w}}_{1,1} & \cdots & {\tilde{w}}_{1,N}\\ \vdots & \ddots & \vdots \\ {\tilde{w}}_{M,1} & \cdots & {\tilde{w}}_{M,N} \end{array}]$$

where ${\tilde{w}}_{d,n}$ is word *n* of document *d*, with *n* = 1 … *N* and *d* = 1 … *M*.

The prepared DTM then acts as an input for rolling LDA to summarize and extract meaningful information from the textual data. The complex model is presented with the assistance of mathematical notations which are summarized with their meanings in Table 2.

**Table 2:** Descriptions of the notations used in the model.

Notation	Description
*τ*	The number of days within each window.
*w*_*t*_	Window with size of *τ* days *where* *w* = *w*_0_ … *w*_*T*_ and *τ* is a coefficient customized by the model user
$\alpha^{\left(w_{t}\right)},\beta^{\left(w_{t}\right)}$	Corpus-level parameters in window *w*_*t*_
$\theta_{d}^{\left(w_{t}\right)}$	Topic mixture, a document-level parameter, in each document *d* in window *w*_*t*_
$z_{n,d}^{\left(w_{t}\right)}$	Topic *n* of document *d* in window *w*_*t*_, assuming the number of topics is not fixed
${\tilde{w}}_{n,d}^{\left(w_{t}\right)}$	Word *n* of document *d* in window *w*_*t*_
$N^{\left(w_{t}\right)}$	Total number of words in all the documents in window *w*_*t*_
$M^{\left(w_{t}\right)}$	Total number of documents in window *w*_*t*_
$D^{\left(w_{t}\right)}$	A vector denoting all input documents, *d*, in window *w*_*t*_
*K*	Number of topics customized by the model user
$\theta_{d,k}^{\left(w_{t}\right)}$	Probability of topic *k* being in document *d* in window *w*_*t*_
$\beta_{k,n}^{\left(w_{t}\right)}$	Probability of word *n* being in topic *k* in window *w*_*t*_;
$\Theta^{\left(w_{t}\right)}$	An $M^{\left(w_{t}\right)}\text{x}K$ matrix of $\theta_{d,k}^{\left(w_{t}\right)}$
$B^{\left(w_{t}\right)}$	A $K\text{x}N^{\left(w_{t}\right)}$ matrix of $\beta_{k,n}^{\left(w_{t}\right)}$
Θ_*t*_	All 4 sets of topic scores generated at time *t*
Θ	A matrix storing all the Θ_*t*_ from *t* = 0 … *T*.

**Fig 2:**
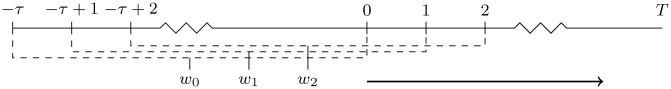
Timeline for rolling LDA modeling.

In rolling LDA, *τ* days of textual data are fitted into the model and the window will roll over from one day to the next window and so on (Fig 2). This approach is similar to the dynamic topic model suggested by [17] for using topic modelling overtime, except that in [17], the discrete time slices in each LDA are all one-year slices (i.e., the window size and the rolling size are both one year). However, in our study, we have a shorter window size and rolling size in order to capture the fast-changing and dynamic market information. Also, it is assumed that the collected textual information consists of many topics where both the topics and the corresponding word components can change overtime. LDA is employed because of its ability to summarize the semantic information from textual data statistically, whilst reducing the dimensionality, as suggested by [32]. The idea of LDA was first introduced in [15]. The LDA model assumes that the documents are probability distribution over the latent topics and the topics are probability distribution over words. According to LDA, new documents are created in three main steps. Firstly, the number of words in the documents is determined, secondly, a topic mixture for the documents over a fixed set of topics is chosen, and finally, a topic mixture is picked on the basis of the multinomial distribution of the documents and a word mixture is then picked on the basis of multinomial distribution of the topics. The additional feature that we have added into the original LDA model is that the LDA model is repeated for each window. The rolling LDA model with a three-level Bayesian hierarchical model is shown below: In LDA, each document d in corpus *D*^(*w*_*t*_)^ is generated by the following process:

1. Choose $N^{\left(w_{t}\right)}∼Poisson(\xi )$.
2. Choose $\theta^{\left(w_{t}\right)}∼Dir(\alpha^{\left(w_{t}\right)})$.
3. For $n=1,\ldots ,N^{\left(w_{t}\right)}$:
  - Choose a topic $z_{n}^{\left(w_{t}\right)}∼Multinomial\left(\theta^{\left(w_{t}\right)}\right).$
  - Choose a word ${\tilde{w}}_{n}^{\left(w_{t}\right)}$ from $p({\tilde{w}}_{n}^{\left(w_{t}\right)}|z_{n}^{\left(w_{t}\right)},\beta^{\left(w_{t}\right)})$, a multinomial probability conditioned on the topic $z_{n}^{\left(w_{t}\right)}.$

The probability of a corpus is defined as below:

$$\begin{array}{c} p(D^{\left(w_{t}\right)}|\alpha^{\left(w_{t}\right)},\beta^{\left(w_{t}\right)})\\ \;\;=\prod_{d=1}^{M^{\left(w_{t}\right)}}\int p\left(\theta_{d}^{\left(w_{t}\right)}|\alpha^{\left(w_{t}\right)}\right)(\prod_{n=1}^{N_{d}^{\left(w_{t}\right)}}\sum_{z_{n,d}^{\left(w_{t}\right)}}p\left(z_{n,d}^{\left(w_{t}\right)}|\theta_{d}^{\left(w_{t}\right)}\right)\;p\left({\tilde{w}}_{n,d}^{\left(w_{t}\right)}|z_{n,d}^{\left(w_{t}\right)},\beta^{\left(w_{t}\right)}\right))d\theta_{d}^{\left(w_{t}\right)} \end{array}$$

Note that the Dirichlet distribution has *K*-dimensions and thus $\theta_{d}^{\left(w_{t}\right)}$ also has *K*-dimensions, where *K* is the assumed number of topics.

**Fig 3:**
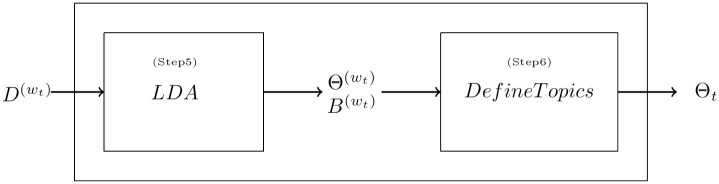
The workflow for rolling LDA modeling.

The documents in each *w*_*t*_ are processed by LDA, and two major outputs are generated, namely, the topic distribution in each document and the word distribution for each topic. The probability distributions are further processed in the *DefineTopics* (i.e Step 6) where we developed ways to connect the output together to capture the evolution of topics overtime. This idea is illustrated in Figs 2 and 3 below.

The two intermediate outputs generated after the LDA process in each window are usually two matrices of the probability of topics for each document and the probability of words for each topic.

$$\Theta^{\left(w_{t}\right)}=[\begin{array}{ccc} \theta_{1,k}^{\left(w_{t}\right)} & \cdots & \theta_{1,K}^{\left(w_{t}\right)}\\ \vdots & \ddots & \vdots \\ \theta_{M^{\left(w_{t}\right)},k}^{\left(w_{t}\right)} & \cdots & \theta_{M^{\left(w_{t}\right)},K}^{\left(w_{t}\right)} \end{array}]\;B^{\left(w_{t}\right)}=[\begin{array}{ccc} \beta_{k,1}^{\left(w_{t}\right)} & \cdots & \beta_{k,N^{\left(w_{t}\right)}}^{\left(w_{t}\right)}\\ \vdots & \ddots & \vdots \\ \beta_{K,1}^{\left(w_{t}\right)} & \cdots & \beta_{K,N^{\left(w_{t}\right)}}^{\left(w_{t}\right)} \end{array}]$$

Later, these two outputs undergo one more process in order to generate the predictors for the final regression model. To achieve this purpose, *k*, which is still an undefined topic at this stage, is defined in the *DefineTopics* section to capture the contextual information of the textual data.

### Define topic measures (Fig 1: Step 6)

In each window *w*_*t*_, there are basically two summarized pieces of information generated by the LDA model, namely, $\Theta^{\left(w_{t}\right)}$ and $B^{\left(w_{t}\right)}$, denoting the probability of topic in document and the probability of word in topic, respectively. With these two sets of probability distributions, a more common way of handling LDA output would be to focus on investigating the semantics meaning of the outputs and trying to label the potential topics, such as in [30, 31]. However, this approach relies heavily on human resources to identify the latent topic and is also subject to human interpretation. Using rolling window setting in rolling LDA, thousands of probability distributions are generated. Therefore, it is essential for us to develop ways of connecting the output together in a systematic way. It is challenging to determine or label what each topic is in each window and to find the linkages within the topics across different windows manually, mainly because over time, the word composition in a topic could evolve and the topics could change. Therefore, instead of assessing the subjective nature of the semantic and contextual information from the words, we introduce four different ways to formulate the topic score measures utilizing both distributions to link the output from the rolling LDA over the period. We call this process as *DefineTopics*.

The first set of topics is labelled according to the chance of occurrence as we are primarily interested in understanding whether a particular topic mentioned often in the documents provides a higher predictive power than topics that are mentioned less frequently. Thus, we consider topics that are more popular to be those latent topics with a higher probability in documents. Topics based on the chance of occurrence are defined as follows:

$$\begin{array}{c} \mu_{k}^{\left(w_{t}\right)}=\frac{\sum_{d=1}^{M^{\left(w_{t}\right)}}\theta_{d,k}^{\left(w_{t}\right)}}{M^{\left(w_{t}\right)}}. \end{array}$$

The result is an ordered-statistics of $\mu_{k}^{\left(w_{t}\right)}$, denoted by ${\tilde{\mu }}_{k}^{\left(w_{t}\right)}$. That is, topic 1 has the largest ${\tilde{\mu }}_{k}^{\left(w_{t}\right)}$, topic 2 has the second largest ${\tilde{\mu }}_{k}^{\left(w_{t}\right)}$ … and topic *K* has the smallest ${\tilde{\mu }}_{k}^{\left(w_{t}\right)}$, i.e. ${\tilde{\mu }}_{1}^{\left(w_{t}\right)}\geq {\tilde{\mu }}_{2}^{\left(w_{t}\right)}\geq \ldots \geq {\tilde{\mu }}_{K}^{\left(w_{t}\right)}$.

Therefore, the first set of topic score measures which we called Popularity is based on the labelled topics where topic *k* at time *t* is defined as $\Theta_{k_{P},t}$, where *k*_*P*_ denotes that topic k is defined by popularity, *P*. Hence, $\Theta_{k_{P},t}$ is defined as equal to ${\tilde{\mu }}_{k}^{\left(w_{t}\right)}$, that is an ordered-statistics, where $\Theta_{k_{P},t}\in \Theta_{t}$. Therefore, $\Theta_{1_{P},t}\geq \Theta_{2_{P},t}\geq \ldots \geq \Theta_{k_{P},t}$, with ${\tilde{\mu }}_{1}^{\left(w_{t}\right)}\geq {\tilde{\mu }}_{2}^{\left(w_{t}\right)}\geq \ldots \geq {\tilde{\mu }}_{K}^{\left(w_{t}\right)}$.

As well as the Popularity measure, we are also interested in understanding the importance of word composition in each topic as a topic is constructed by a set of words and potentially different among topics. So, after the construction of the Popularity measure and given the topics are labelled as 1 to *K* on the basis of chance of occurrence, another set of scores called Word Diversity is constructed using the word distributions. In other words, both Popularity and Word Diversity have the same order in terms of chance of occurrence (i.e. popularity). However, Word Diversity takes into the account of the diversity of the topic composition. For example, a topic related to national security could be constructed by words such as “terrorism,” “war,” and “sanctions,” with corresponding weights. If the weights of the three words is similar (e.g., 33% for each word), the topic is defined as diverse. To create a diversity measure, we adopted a concept similar to the Gini diversity in a decision tree model, that is

$$Gini\;Diversity=1-\sum_{i=1}^{N}p_{i}^{2},$$

where *p*_*i*_ is the probability of the appearance of each word and *N* is the total number of words in a topic. However, if we apply this measure directly to our dataset, the numeric value of the diversity measures of the words would all be close to one, with very little difference between each word as the *N* is normally extremely large. This insignificant changes over time can easily lead to an insignificant effect on the regression response. To refine this measure, we break the constraint limiting the value to be between 0 and 1 by adopting the following formula so that the smaller the value, the more diverse the words are in a topic.

$$\begin{array}{c} Diversity\;Measure=\sum_{i=1}^{N}{\left(\sqrt{N}p_{i}\right)}^{2} \end{array}$$ (1)

Hence, the second set of topic score measures, Word Diversity, shows the word diversity of topic *k* at time *t* and it can be denoted as $\Theta_{k_{W},t}$, with *k*_*W*_ meaning that topic *k* is defined by word diversity, *W*. According to Eq 1, $\Theta_{k_{W},t}$ is defined as below and $\Theta_{k_{W},t}\in \Theta_{t}$:

$$\begin{array}{c} \Theta_{k_{W},t}=\sum_{n=1}^{N^{\left(w_{t}\right)}}{\left(\sqrt{N^{\left(w_{t}\right)}}\beta_{k,n}^{\left(w_{t}\right)}\right)}^{2} \end{array}$$ (2)

where $\beta_{k,n}^{\left(w_{t}\right)}$ is the probability of word *n* being in topic *k* in window *w*_*t*_.

The weights can change over time, and we assume word diversity to be one of the factors affecting the market volatility: for example, if “terrorism,” “war,” and “sanctions” have the same weight, it could imply that no major events are occuring, but if one of the words suddenly dominates the topic, this could imply a sudden event that may affect the stock market. This is why we should consider word diversity as one of the variables in predicting volatility. In addition, looking at the word diversity of popular and non-popular topics, a measure reflecting purely whether the diversity of words can affect volatility, should also be considered. Thus, as well as labelling topics by popularity, word diversity should also be taken into account in labelling the topics. In this way, we can identify whether the topics with more diverse word composition predict market volatility better than those with less diversity. Therefore, a new ordered-statistics of $\Theta_{k_{W},t}$, which is denoted as ${\tilde{\Theta }}_{k_{W},t}$, is proposed on the basis of Eq 2: that is, topic 1 is re-defined as the topic with the largest word diversity, ${\tilde{\Theta }}_{k_{W},t}$; topic 2 has the second largest ${\tilde{\Theta }}_{k_{W},t}$ and topic *K* has the smallest ${\tilde{\Theta }}_{k_{W},t}$. Therefore, the third set of topic score measures that we called Diversity-ranked Word Diversity is based on the ordered-statistics of word diversity, $\Theta_{k_{C},t}$, where *k*_*C*_ means that topic *k* is defined by diversity-ranked word diversity, *C*, where ${\tilde{\Theta }}_{k_{C},t}\in \Theta_{t}$, and ${\tilde{\Theta }}_{1_{C},t}\geq {\tilde{\Theta }}_{2_{C},t}\geq \ldots \geq {\tilde{\Theta }}_{K_{C},t}$.

The final set of topic scores is called Topic Diversity, a similar concept to Word Diversity at document level. A document is composed of various topics and we believe that if a topic suddenly dominates, or if no topic dominates, there may be an event affecting market volatility. Therefore, it is also intriguing to look at how Topic Diversity can measure the balance of each topic in every document and predict volatility. Therefore, the fourth set of topic score measures is called Topic Diversity where it is computed by measuring how evenly distributed the probability distribution of the topics for each document is. Topic Diversity refers to at time *t*, is denoted as Θ_*E*,*t*_, with *E* meaning that this set of topic scores is defined by topic diversity, *E*. Θ_*E*,*t*_ is defined as below according to Eq 1, where Θ_*E*,*t*_ ∈ Θ_*t*_:

$$\begin{array}{c} \Theta_{E,t}=\frac{\sum_{d=1}^{M^{\left(w_{t}\right)}}\left(1-\sum_{k=1}^{K}\theta_{d,k}^{\left(w_{t}\right)}\right)}{M^{\left(w_{t}\right)}} \end{array}$$

### Rolling window regression (Fig 1: Step 7)

**Fig 4:**
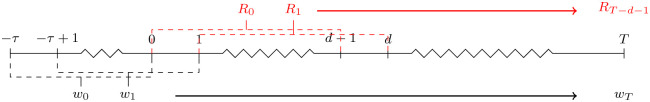
Timeline for rolling regression model.

The four topic score measures introduced are essentially the regressors to predict the market volatility measures. In the following section, we explain how we use these topic measures and study their predictive performance in the prediction model. We adopted regression as our prediction model in which rolling window regression is implemented. The same rolling window concept is applied to the regressions as shown in Fig 4, but with different window sizes. Different window sizes (e.g. 30, 60 and 90 days) were implemented during the experiment. For illustration purposes, *R*_*t*_, indicating the regression window starting at time *t*, is added to the timeline in addition to the rolling window *w*_*t*_ used in rolling LDA. The new rolling window process for the regression model, in which the window starts from *t* = 0 is coloured in red in Fig 4 For example, *R*_0_ from *t* = 0 till *t* = *d* − 1 is the first regression period, where we would use regressors in *R*_0_ to predict the value at *t* = d. In other words, if the window size is set as 30 calendar days, then *d* = 31 days. The window keeps rolling until the first data point of the window to start at *T* − *d* − 1, yielding a prediction at *T*.

In a single rolling window regression, *R*_*t*_, the set of predictors used in *R*_*t*_, $X^{\left(R_{t}\right)}$, and the responses in *R*_*t*_, $y^{\left(R_{t}\right)}$, are defined as below:

$$y^{\left(R_{t}\right)}=[\begin{array}{c} y_{t}\\ \vdots \\ y_{t+d-1} \end{array}]\;X^{\left(R_{t}\right)}=[\begin{array}{cccc} \Theta_{1_{a},t-1} & \cdots & \Theta_{K_{a},t-1} & \Theta_{E,t-1}\\ \vdots & & \ddots & \vdots \\ \Theta_{1_{a},t+d-2} & \cdots & \Theta_{K_{a},t+d-2} & \Theta_{E,t+d-2} \end{array}]$$

where, *a* ∈ {*P*, *W*, *C*}, and therefore, **X** includes all sets of topic scores generated in all rolling windows. Before applying the topic scores to the regression model, a logit transformation of **X** is performed. As the nature of all the sets of topic scores is probability, the data points tend to be concentrated at 0 and packed together within a range. Hence, logit transformations help to increase the variations of the data points in each set of topic scores and enhance the performance of the regression. After the transformation, “best subset” is used to filter out useful predictors. These topic scores are grouped into subsets, where each subset is denoted as *i*, and fitted into the regression model, as depicted below, (i.e. Eq 4). In addition to the topic scores, the rolling means, ${\bar{y}}^{\left(R_{t}\right)}$, (calculated in Eq 3), are also considered in the model to obtain a fair comparison of the performance with the benchmark model, which is a regression model with the rolling mean as the predictor (refer to Eq 6 for more details).

$$\begin{array}{c} {\bar{y}}_{t}=\frac{\sum_{i=1}^{d}y_{t-i}}{d} \end{array}$$ (3)

where, ${\bar{y}}^{\left(R_{t}\right)}$ = $\left\{{\bar{y}}_{t}\;...\;{\bar{y}}_{t+d-1}\right\}$

The rolling window regression process is defined as below:

For each regression window *R*_*t*_, each subset of $X^{\left(R_{t}\right)}$, i.e. $X_{i}^{\left(R_{t}\right)}\in X^{\left(R_{t}\right)}$, will be fitted into the regression model, (i.e. Eq 4):

$$\begin{array}{c} y^{\left(R_{t}\right)}=\alpha_{i}^{\left(R_{t}\right)}+{\bar{y}}^{\left(R_{t}\right)}+X_{i}^{\left(R_{t}\right)}\beta_{i}^{\left(R_{t}\right)}+\epsilon^{\left(R_{t}\right)} \end{array}$$ (4)

In each subset regression, $R_{i}^{2}$ from Eq 4 can be calculated. The model with the highest $R_{i}^{2}$, which is generated by the *i*^*th*^ subset of predictors will be chosen as the topic score model for predicting the market volatility. This algorithm is known as the best subset regression and tends to perform better than other model selection algorithms [33]. The prediction process using the chosen model can then be defined as follows:

$$\begin{array}{c} {\hat{y}}_{t+d}={\hat{\alpha }}_{i}^{\left(R_{t}\right)}+{\bar{y}}_{t+d-1}+X_{i,t+d-1}{\hat{\beta }}^{\left(R_{t}\right)} \end{array}$$ (5)

where, ${\hat{y}}_{t+d}\in \hat{y}$; *R*_*t*_ = regression window at time t, with a size of *d* calendar days, where *t* = 0 … *T* − *d* − 1; $\beta_{i}^{\left(R_{t}\right)}$ = coefficient of the corresponding predictor, $X_{i}^{\left(R_{t}\right)}$ where *i* = 1 … $C_{s}^{Z}$; *Z* is the total number of predictors in $X^{\left(R_{t}\right)}$; and thus $C_{s}^{Z}$ is the total number of combinations containing subset size of *s*.

**Fig 5:**
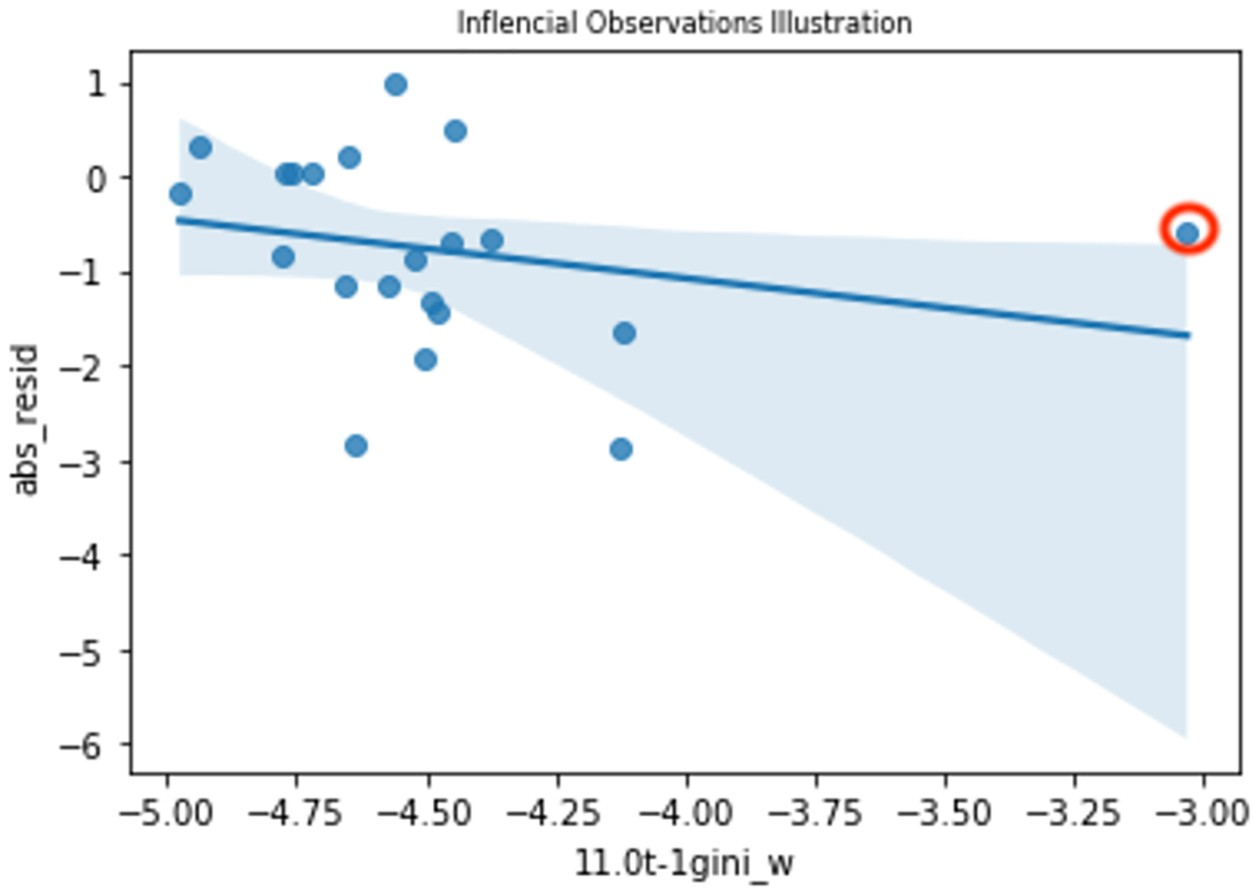
Illustration of influential observations.

This model fitting and predicting process keeps going until the regression window, *R*_*T*−*d*−1_, generates all prediction outputs, $\hat{y}$, containing predictions $\{{\hat{y}}_{t+d}\;...\;{\hat{y}}_{T}$. However, this simple rolling regression model may not perform well because the performances of some regressions in some windows *R*_*t*_ can be limited by outliers, extrapolation problems in prediction, and overfitting, especially if there is a relatively small sample size in each window. For example, the effect on the model fitting caused by outliers, or by influential observations, is illustrated in Fig 5, showing the fitted regression line using one of the predictors with an influential observation in one regression window.

The circled data point is the influential observation that leveraged the regression line to a certain angle. Removing that data point obviously makes the slope steeper and obtains a regression line that is better fitted with the other data points if they are fitted into Eq 4. Due to the adoption of a relatively small window size for each regression (e.g. 1–3 months), this phenomenon is very likely to appear because financial markets as well as the topic scores always fluctuate more in a short timeframe. To address this issue, those extreme data points should be identified and removed from each subset regression in each window. Therefore, Cook’s Distance (COOKD) from [33] is adopted in this paper. Observations with a calculated COOKD ≥ 1 are identified as the influential observations and thus, are eliminated. The remaining observations are fitted into the regression model in Eq 4 again and a new $R_{i}^{2}$ is calculated. Besides affecting the model-fitting (i.e. Eq 4), having a short timeframe will also make the prediction process unreasonable if the predictor adopted in Eq 5 has an extreme value. For example, if the predictor, *X*_*i*,*t*+*d*−1_, has a value like the x-coordinate value of the red point in Fig 5, that makes itself deviate from the group of fitted data, $X_{i}^{\left(R_{t}\right)}$, then the regression line is extrapolated, causing the prediction to be inaccurate. To avoid this problem, a rule is applied in the prediction process (i.e. Eq 5) to ensure each value in *X*_*i*,*t*+*d*−1_ is within *range*. If this rule is not satisfied in a model, another model with a different subset of predictors should be adopted. The *range* has an upper bound and a lower bound constructed by the maximum and minimum values of each column of the array, $X_{i}^{\left(R_{t}\right)}$ respectively. For example, if the number of predictors in $X_{i}^{\left(R_{t}\right)}$ in Eq 4 equals to 2 (i.e. number of columns = 2), then the value in the first column of *X*_*i*,*t*+*d*_ in Eq 5, denoted as $X_{col_{1},i,t+d}$, should follow the following condition: $min\left(X_{col_{1},i}^{\left(R_{t}\right)}\right)\;\leq \;X_{col_{1},i,t+d}\;\leq \;max\left(X_{col_{1},i}^{\left(R_{t}\right)}\right)$, where $X_{col_{1},i}^{\left(R_{t}\right)}$ means the values in column one of $X_{i}^{\left(R_{t}\right)}$ in Eq 4. The same applies for the second column (i.e. *col*_2_).

**Fig 6:**
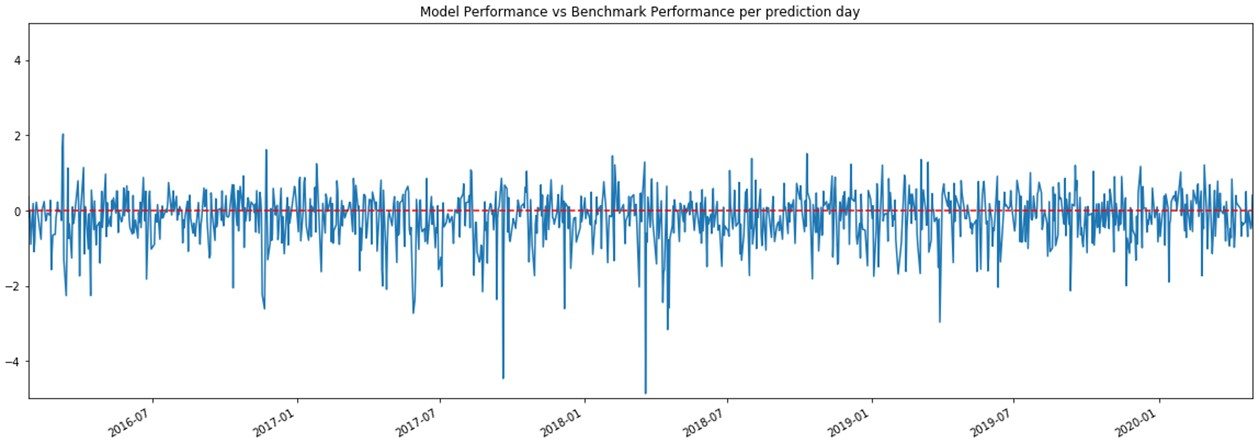
Model performance vs benchmark performance per prediction day. The overall performance comparison between the chosen topic model and benchmark model measured by RMSE each day. Positive peaks indicates the topic model outperformed the benchmark model and vice versa.

With a small timeframe in the fluctuating financial market, overfitting is also another common problem that may result in extreme predictions, or having predictions that are much worse than the benchmark model. Fig 6 is an example of the consequence of overfitting. A positive peak means that the prediction value of the topic score model is closer to the actual value than the benchmark model, while a negative trough indicates that the benchmark model has a higher accuracy. It can be seen that there are some extreme predictions (extremely negative troughs) at some times, indicating that topic scores in the model result in far more unreasonable predictions than having no topic scores in the model. To avoid extreme predictions, one option is to set a rule that is again a boundary, for the predicted value. This is similar to setting a boundary for predictors (i.e. *range* is set with respect to the corresponding column in the array). Since there is only one column of response, it means that the rule is $min\left(y^{\left(R_{t}\right)}\right)\;\leq \;{\hat{y}}_{t+d}\;\leq \;max\left(y^{\left(R_{t}\right)}\right)$. Again, if this rule is not satisfied in a model, another model with a different subset of predictors should be adopted.

Fig 6 also implies that the topic score model may have a chance of performing better than the benchmark in some periods (positive peaks), while performing worse than the benchmark model in other periods(negative troughs). In other words, the topic score model performs better when it is restricted to some condition. Here, the condition can be hypothesized as the in-sample fitting confidence (i.e. model *R*^2^). If the confidence is high enough, that is higher than a *threshold*, the prediction from the topic score should be adopted; otherwise, the prediction from the benchmark model, as defined as below, should be adopted.

$$\begin{array}{c} y^{\left(R_{t}\right)}=\alpha_{i}^{\left(R_{t}\right)}+{\bar{y}}^{\left(R_{t}\right)}+\epsilon^{\left(R_{t}\right)} \end{array}$$ (6)

$$\begin{array}{c} {\hat{y}}_{t+d}=\alpha_{i}^{\left(R_{t}\right)}+{\bar{y}}_{t+d-1} \end{array}$$ (7)

Eqs 6 and 7 are known respectively as the benchmark model fitting function and the benchmark predicting function.

**Fig 7:**
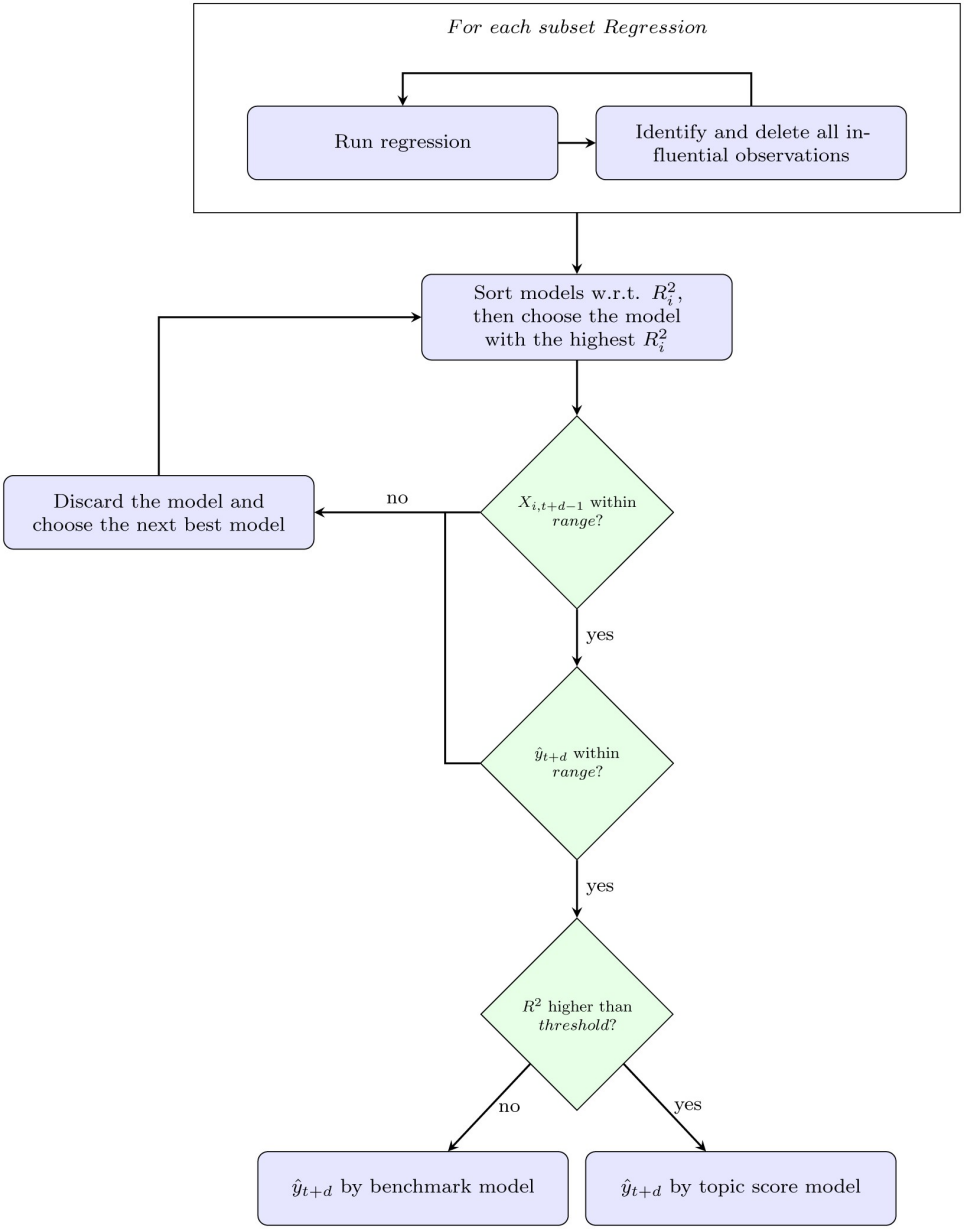
Regression flow chart. Decision rules for whether to choose the benchmark model or topic score model for prediction.

All of the solutions to the potential problems discussed above act as tuning parameters and are organized into step-by-step decision rules, as depicted in Fig 7, reflecting the mechanism of how the final prediction comes out in each window *R*_*t*_.

The prediction performance obtained from the best subset regressions are evaluated against the benchmark model. Since the prediction approach adopted is a hybrid model approach where only topic models with a threshold higher than a certain *R*^2^ are used, the rest will choose the benchmark model for the prediction. Therefore, we focus on the performance when the topic model is used. The predicted datapoint when the topic model is used is denoted as ${\hat{y}}_{t^{*}}$ where ${\hat{y}}_{t^{*}}\in \hat{y}$ and t* means the time *t* when our model is chosen over the benchmark model for the prediction. T* is then denoted as the total number of times that our model is chosen over the benchmark model. To evaluate the performance of our model, various measures are calculated on the basis of ${\hat{y}}_{t^{*}}$; these measures are detailed below.

The first measure is a conditional root mean square Error(RMSE). The RMSE formula is shown as follows:

$$\begin{array}{c} RMSE_{cond}=\sqrt{\frac{\sum_{t^{*}=1}^{T^{*}}{\left(y_{t^{*}}-{\hat{y}}_{t^{*}}\right)}^{2}}{T^{*}}} \end{array}$$ (8)

The second measure is conditional mean absolute error(MAE). Although MAE normally provides similar results to RMSE, in this case, MAE is particularly useful because it can prevent the extreme results predicted by our model from being dominating the performance. The MAE formula is shown as follows:

$$\begin{array}{c} MAE_{cond}=\frac{\sum_{t^{*}=1}^{T^{*}}\left|\left(y_{t^{*}}-{\hat{y}}_{t^{*}}\right)\right|}{T^{*}} \end{array}$$ (9)

The above two measures can show how well a single model performs. However, in order to provide a clearer picture of whether our model is better or worse than the benchmark model, we can calculate the number of occurrences (as a percentage) where our model has a lower *RMSE*_*cond*_ and *MAE*_*cond*_ than the benchmark model. Hence, the third measure is the conditional probability that the topic model is performing better than the benchmark model. When the conditional probability is larger than 0.5, given the topic model is chosen because of its acceptable in-sample performance, it means the topic model performs better than the benchmark model over 50% of the time. This is a good sign indicating that the topic model is useful, while a conditional probability lower than 0.5 is a bad sign. Moreover, in order to quantify the good or bad signs, we can even take a ratio between the performance measures of the predictions from our model, ${\hat{y}}_{t^{*}}|model$, and the predictions from the benchmark model, ${\hat{y}}_{t^{*}}|bench$, which are calculated from Eqs 5 and 7 respectively. As a result, we can obtain the fourth measure, the conditional RMSE ratio, as well as the fifth one, the conditional MAE ratio. The respective calculations are shown below:

$$\begin{array}{c} RMSE_{ratio}=\sqrt{\frac{\sum_{t^{*}=1}^{T^{*}}{\left(y_{t^{*}}-{\hat{y}}_{t^{*}}|bench\right)}^{2}}{\sum_{t^{*}=1}^{T^{*}}{\left(y_{t^{*}}-{\hat{y}}_{t^{*}}|model\right)}^{2}}} \end{array}$$ (10)

$$\begin{array}{c} MAE_{ratio}=\frac{\sum_{t^{*}=1}^{T^{*}}\left|\left(y_{t^{*}}-{\hat{y}}_{t^{*}}|bench\right)\right|}{\sum_{t^{*}=1}^{T^{*}}\left|\left(y_{t^{*}}-{\hat{y}}_{t^{*}}|model\right)\right|} \end{array}$$ (11)

## Empirical study

In this empirical study, the entire process, including the kinds of data are collected and how the data are processed and fitted into the model, is discussed following the flow chart in Fig 1, with the aim of finding out how textual information can help to predict the market volatility. In this experiment, all of the steps in the flow chart are illustrated and discussed, and the findings yielded from this experimental settings are presented. The entire experiment is supported by a MacBook Pro 2019, with a 1.4 GHz Quad-Core Intel Core i5 processor, and a 16 GM 2133 MHz LPDDR3 memory.

### Textual data collection (Fig 1: Step 1)

**Fig 8:**
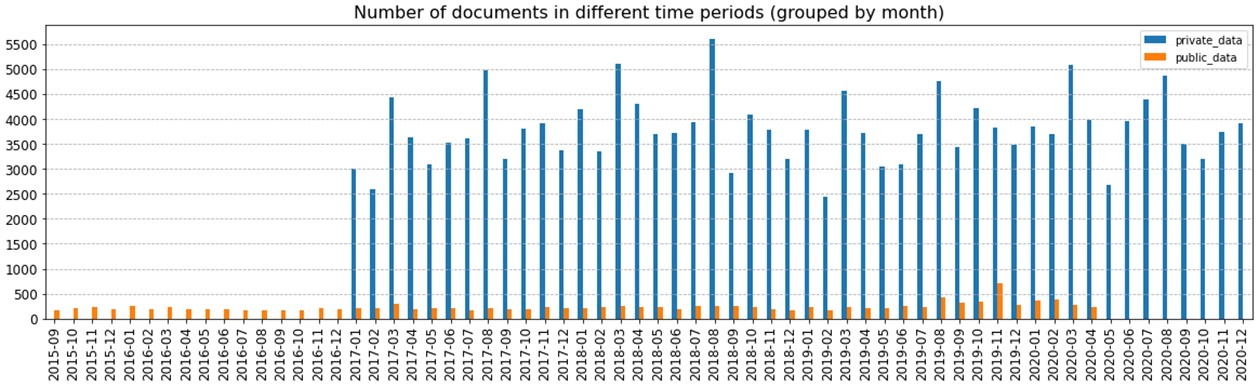
Number of documents per month in the public and private data respectively.

In this study, there are two different datasets collected. We would consider news from open sources as the public data and news from a licensed database as the private dataset. The public data is news related to finance, economy, and business, collected through various open sources (i.e. Reuters, Hong Kong Business, and CNBC). The advantage of using open sources for the study is that they are easily accessed by the public. However, compared to some paid news sources, these news sources may have less information concerning the market movement as the main audience is the general public. The news items are all scraped by Requests and BeautifulSoup in Python, and the combined corpus consists of 14,364 documents from 2015–09-09 to 2020–04-30, after removing some news with no content and believed to be non-recoverable. The corpus of private data consists of more than 182,000 documents from 2017–01-01 to 2020–12-31 from a Reuters licensed database. An overview of the number of documents per month in the two datasets is also presented in Fig 8. The number of documents per month is quite evenly distributed and there are on average more documents in the private data set in blue (3794 documents per month) than the public data set in orange (234 documents per month).

### Data pre-processing and pre-filtering (Fig 1: Step 2)

As the financial data are mostly well organized, only the textual data cleansing process is discussed in detail. The data cleansing process plays a very important role in textual data analysis as the amount of information given from texts can easily be ignored due to the noises. In this study, the entire data cleansing procedure applying to both datasets is split into two steps: basic data cleansing and further data cleansing. As with [3], the entire basic cleansing procedure is carried out by the Natural Language Processing Toolkits (NLTK) library in Python. The algorithm is presented as below:

1. Remove all unnecessary string patterns; for example, headers, news reporter names, words inside parentheses (mostly ticker names), words inside square brackets, (mostly ID names), and dollar signs (e.g. HKD, USD)
2. Remove all punctuations, stop words, and self-defined frequent words such as “weekdays” or “percent”
3. Transform the entire corpus to lower case
4. Lemmatize the corpus using WordNetLemmatizer

After the initial data cleansing process, a DTM is extracted as the output. The cleaned data in the form of a DTM are then put into the rolling LDA model to generate the topic scores that are the final predictors of the rolling regression model. However, this sizable matrix still contains a lot of noises, such as frequent words and rare words, that drag down the efficiency of the rolling LDA model. Therefore, further data cleansing is performed to solve the issue. This procedure is implemented through a trial-and-error process in the rolling LDA procedure. First, in each window, *w*_*t*_ in rolling LDA, a cross-validated grid search is applied to find the filtering criterion. In addition, by assessing the result of the model printing out the words comprising a topic, a criterion is claimed to be adoptable if the word-mixture in each topic is mostly different (implying no domination from frequent words). The following filtering criterion is finally adopted: ignore words that appear either in more than 30% of the documents or in less than 0.1% of the documents in each window for public data and ignore words that appear either in more than 20% of the documents or in less than 0.1% of the documents for private data. The quality of the data after the entire data cleansing process inside the rolling LDA is presented in the appendix. Fig 18 in S1 File shows the distribution of the length of document. As expected, after the data cleansing process, the private data tend to have far fewer words left inside each document in general. A reasonable explanation is that the private dataset is more organised as it comes from a single source whilst the public dataset is obtained using data from different sources and therefore is more difficult for data cleansing. Fig 19 in S1 File also shows the uniqueness of each word in its corresponding dataset. As expected, as the two datasets contain news of a few years and both of them have a substantial number of documents, the word uniqueness appears to be very high. In total, around 25,000 words were processed in LDA from the private data whilst 21,000 words were processed in LDA from the public data.

### Financial data collection (Fig 1: Step 3)

The HSI, representing the performance of the Hong Kong financial market, is chosen to allow the analysis to reflect the overall economy. Hence, the daily closing price of the HSI, downloaded from Yahoo Finance, is used. The period of this dataset is from 2015–09-09 to 2020–12-31, giving 1,480 observations. The financial data is applicable to both textual datasets. A summary of the data collected is shown in Table 3.

**Table 3:** Description of the data collected.

Data Type	# of observations	Timeframe	Sources	Description
*Textual*(*public*)	14,364	2015–09–09 to 2020–04–30	Open sources: Reuters, Hong Kong Business, CNBC	News related to finance, economy and business
*Textual*(*private*)	182,124	2017–01–01 to 2020–12–31	Licensed Reuters database	News related to Hong Kong
*Financial*	1,480	2015–09–09 to 2020–12–31	Yahoo! Finance	Closing price of Hang Seng Index (HSI)

### Risk modelling (Fig 1: Step 4)

**Fig 9:**
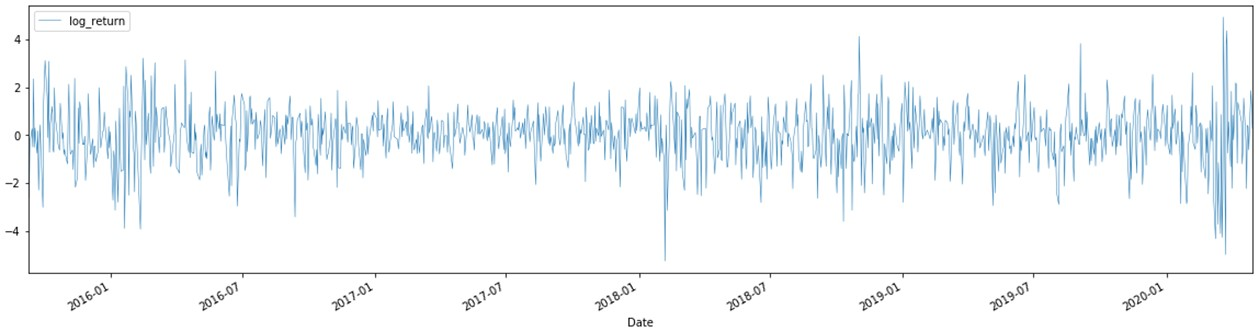
Log return of the HSI.

**Fig 10:**
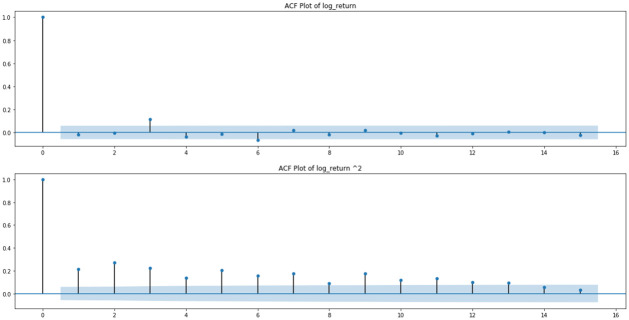
ACF plot of log return and squared log return of the HSI.

**Fig 11:**
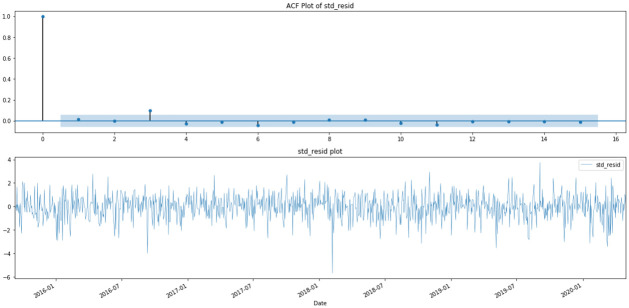
ACF and time-series plot of standardized GARCH residual.

The response of the regression model, the market volatility, or the market risk, is formed by extracting the standardized residual of a GARCH(1,1) model of the HSI. The reason for adopting a GARCH model is that it helps to explain the volatility using the time series data as the only predictor, so what is left behind as the non-explained part is the volatility that can only be explained by other measures [34]. Hence, the GARCH residual would be ideal enough to test whether topic scores are good measures to explain the volatility which has not been explained by the index on its own. The process by which the HSI closing price can be formulated into a risk measure is discussed below. First, the log return, *R*_*t*_, of the HSI is calculated and some visualizations of the return are performed (see Figs 9–11). In graphs Figs 9 and 10, volatility clustering and the AR process for squared log return can be observed. As these properties fit the GARCH process, they are tested to fit into the GARCH(1,1) model. As indicated in graph Fig 11, GARCH(1,1) is an appropriate GARCH process and thus, the corresponding standardized GARCH residual, *r*_*t*_, is taken as the risk measure. The entire process is denoted below.

$$\begin{array}{c} R_{t}=W_{t}\sqrt{\omega +\beta \sigma_{t-1}^{2}+\alpha R_{t-1}^{2}} \end{array}$$ (12)

$$\begin{array}{c} r_{t}=\frac{R_{t}}{\sigma_{t|t-1}} \end{array}$$ (13)

where, *R*_*t*_ is the log return of the adjusted closing price of the HSI at time t, $\sigma_{t-1}^{2}$ is the variance of the HSI at time t − 1, *σ*_*t*|*t*−1_ is the conditional volatility of the HSI; and *W*_*t*_ is the white noise at time t.

Rather than the standardized GARCH residuals, the log of the absolute standardized GARCH residuals are adopted for the following analysis because they are a better measure for risk as they measure purely the magnitude of fluctuation in the market. It will act as a volatility proxy in the financial market.

### Retrieve textual information by rolling LDA and define topic measures (Fig 1: Steps 5 and 6)

With regards to how we choose the model setting for rolling LDA, the cleaned DTM is ready to input into the rolling LDA after the data cleansing and pre-filtering process with the filtering criterion of removing words that appear in more than 30% or 20% of the documents for public data and private data respectively, or in less than 0.1% of the documents in each window. This effectively reduces the dimension of the DTM without losing much information. We adopted the window size, *τ*, of 30 days and the rolling size of one day. The reason is that we are trying to strike a balance between capturing the dynamic or diverse nature of the textual information in the news and having a sufficient sample size for the modelling.

The only hyper-parameter input for the rolling LDA model, which is the number of topics, which we assume it to be *K* = 15, as a grid search conducted to find out the optimal number of topics in each window produced results ranging from 6 to 14; therefore, we assume 15 to be a reasonable input. Fixing the number of topics allows the output, such as topic distribution, to be more comparable across periods. The rolling LDA model takes around seven hours to process all 180,000 documents divided into 965 periods in the private data. The public data with around 15,000 documents divided by 1118 periods, it takes only one hour to finish the LDA process.

### Rolling window regression (Fig 1: Step 7)

Both the response and the predictors have been generated at this stage. In summary, the response is the absolute standardized GARCH residuals, while the predictors are four sets of topic scores, containing 46 individual predictors, where each set contains 15 topics (except for Topic Diversity, where there is only one topic). Hence, the response and the predictors are fitted into the rolling regression model. To maintain the computing efficiency, in the best subset regression of each window, only the best subset of one (*model* 1) and two (*model* 2) is chosen (i.e. there are $C_{1}^{46}$ and $C_{2}^{46}$ combinations, respectively). Furthermore, the interaction term of the two predictors selected by the best subset of two is also considered, and this best subset of two with its interaction term is labelled *model* 3. The expressions of the three different models are summarized in Table 4. The regression model generally takes less than an hour and a half to run *model* 3 with a window size of 30 for both private and public data with around 1000 days of observations. *Model* 2 shares a similar time consumption but *model* 1 takes only a few minutes to complete a specific window size. However, for experimental purposes, we processed three different models with three different window sizes.

**Table 4:** Model settings of the empirical study.

Model Name	# of Topic Scores Predictors	# of Combinations in Best Subset Regression
*model* 1	1 topic score measure	$C_{1}^{46}$
*model* 2	2 topic score measures	$C_{2}^{46}$
*model* 3	2 topic score measures + interaction terms	$C_{2}^{46}$

## Main results

**Fig 12:**
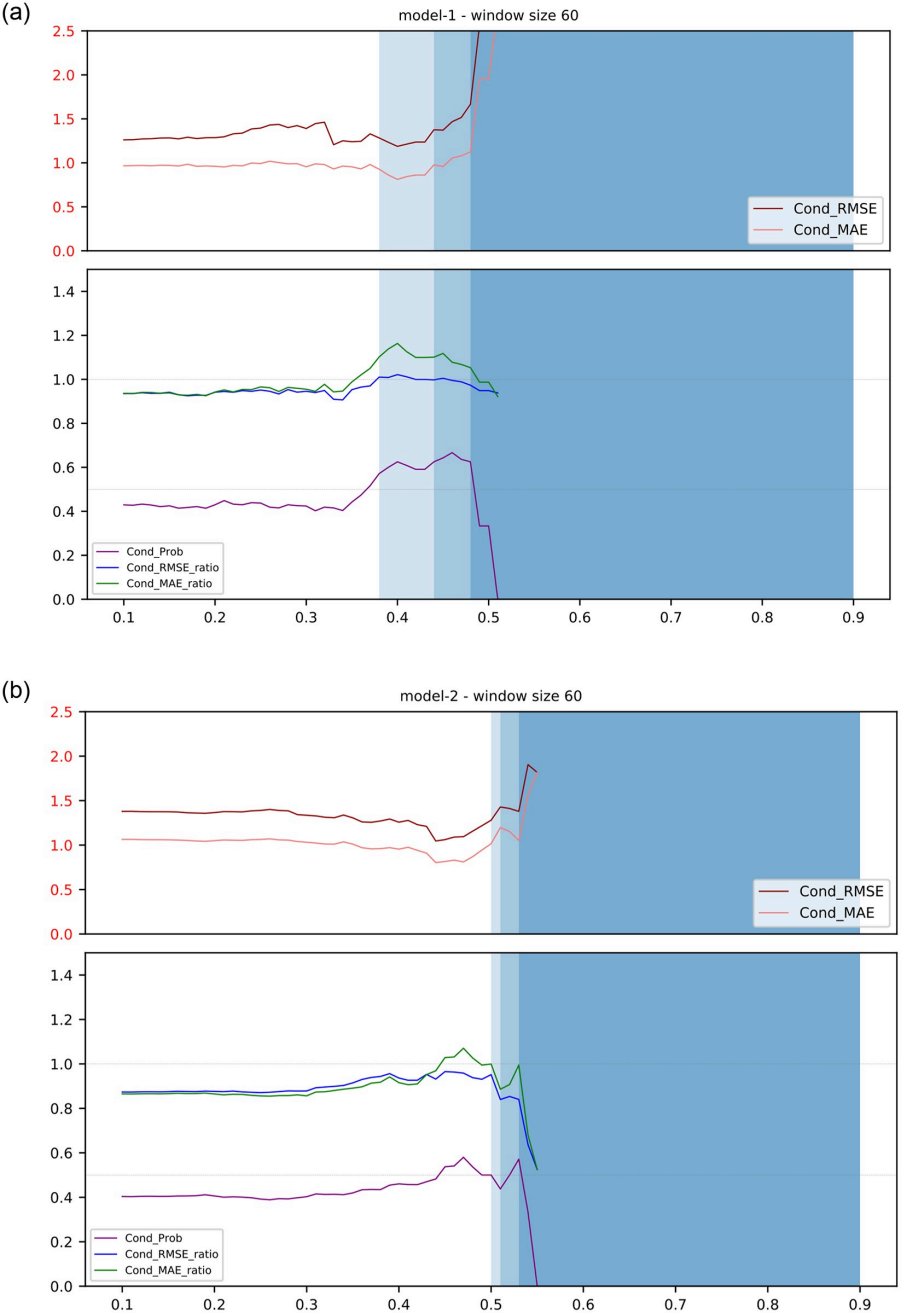
The graphs represent the two best models generated from the public data and the private data, respectively. The y-axis represents the value of the evaluation metrics whilst the x-axis represents the *threshold*. The top graph in each diagram shows the conditional RMSE and the conditional MAE, and the bottom graph shows the RMSE ratio, the MAE ratio, and the conditional probability. The shaded area indicates the number of days that the topic model was chosen. The darkest blue, mid blue, the lightest blue and white areas represent 0–10, 10–20, 20–30 and above 30 observations, respectively. (a) Best model for the public data. (b) Best model for the private data.

In this section, the performances of both the public and private data are discussed. Each of the results are organised into a figure with nine graphs, representing three different models (i.e., model 1, model 2 and model 3) in three different window sizes (i.e., 30, 60 and 90 days). Each graph depicts the performance of the hybrid model approach we proposed above in predicting the volatility proxy, that is, the log of the absolute standardized GARCH residuals of the HSI using different performance measures with different regression window sizes. The performance indicators are as discussed in the Methodology section: conditional RMSE, conditional MAE, conditional probability, conditional RMSE ratio, and conditional MAE ratio (Eqs 8–11). From all the tested model, we have chosen the best model from the public data (i.e., model 1—window size 60) as well as the best model from the private data (i.e., model 2—window size 60) for comparison (Fig 12), whilst the other tested models are placed in **Supporting information** for reference (i.e., Fig 20 in S1 File for the public data results and Fig 21 in S1 File for the private data results).

These two models are considered to be the best among all the models in public and private data because both the conditional MAE ratios are larger than 1 and the conditional probabilities are larger than 0.5 at some specific thresholds. In addition, another factor for determining the best model is the proportion of days or the number of observations where the topic score model is chosen over the benchmark model in our hybrid model approach because it would be less effective to evaluate the performance between the topic score model and the benchmark model if there are too few observations. Therefore, we have chosen the specific thresholds that satisfy all the requirements. Table 5 shows the details for each evaluation metric which is mentioned in **Methodology** and the specific threshold for each model.

**Table 5:** Different performance evaluation measures for the best models of public and private data.

Model	Conditional RMSE	Conditional MAE	Conditional RMSE ratio	Conditional MAE ratio	Conditional Probability	Predicted days	Total days
**Public data** (model 1 with window size of 60 at threshold 0.4)	1.1874	0.8119	1.0216	1.1633	0.6250	24	1032
**Private data** (model 2 with window size of 60 at threshold 0.45)	1.0901	0.8152	0.9655	1.0287	0.5373	67	913

**Fig 13:**
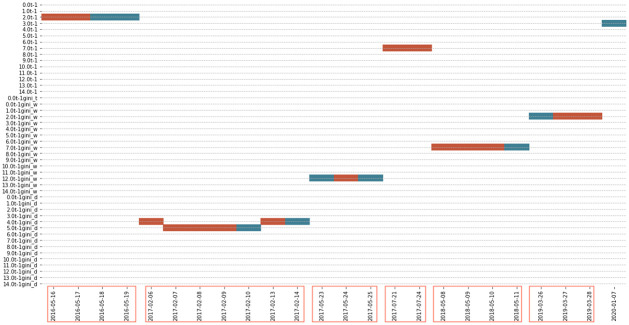
Different topic score models chosen for prediction in model 1 in public data. The graph shows the result from the best model of the public data—model 1 under the window size of 60 days, with a threshold of 0.4. It indicates the topic score measures chosen by the best subset selection for the predictions and dates in the x-axis representing the last day of the regression period. The red (blue) colour represents where the topic score (benchmark) model outperformed the benchmark (topic score) model.

The statistics in Table 5 show the best model of the public data (i.e., model 1 with window size of 60 at threshold 0.4) performs better than the benchmark model in the 24 days where topic score model is adopted, from the total number of 1032 days. Looking at the conditional probability, we also observe that within those 24 days, the topic score model performs better than the benchmark in about 62% of the days. The conditional MAE ratio also shows that the benchmark tends to have 16% more prediction errors in these 24 days. In additional to the model performance, we also looked for any other noticeable observations in those prediction days. Fig 13 shows that the 24-day observations can be clustered into seven periods. This implies that the topic score models can usually be applied in a few consecutive days. This may be due to some critical events affecting volatility during those prediction days, but unfortunately, these events are not significant enough for us to identify them in our corpus.

**Fig 14:**
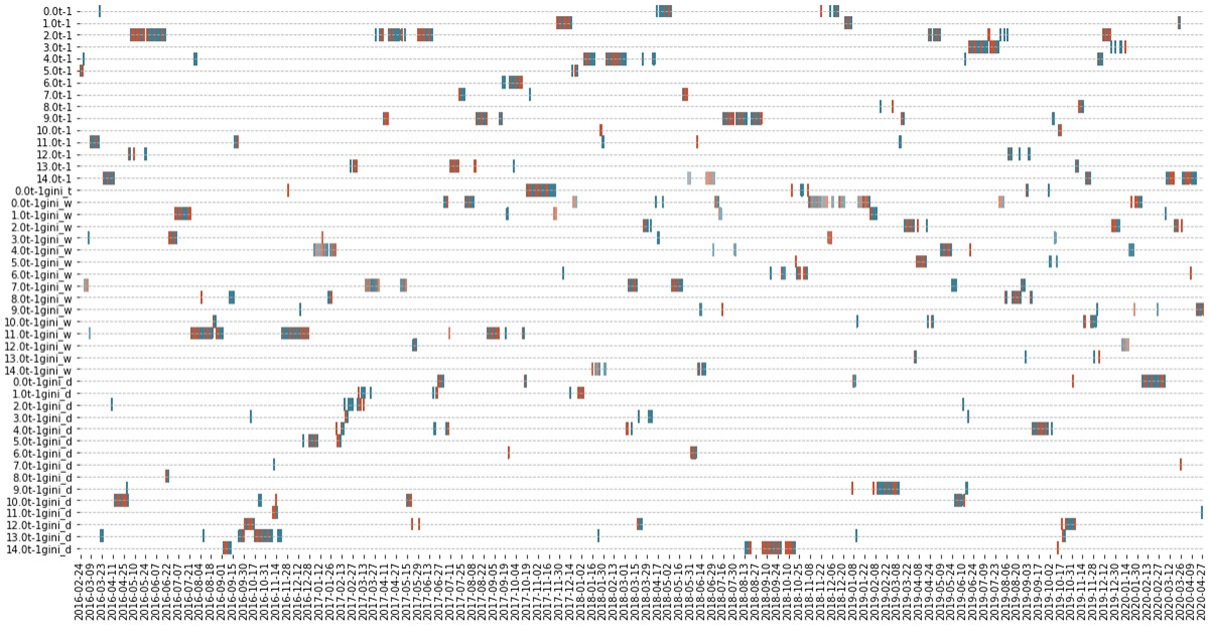
Different topic score models chosen for prediction in model 1 when threshold setting = 0. This graph shows the regressors chosen from model 1 with the window size of 60 days, assuming the threshold = 0. In the y-axis, the regressors 0.0t-1 to 14.0t-1 represent the Popularity measures, 0.0t-1gini_t represents the Topic Diversity measures, 0.0t-1gini_w to 14.0t-1gini_w represent Word Diversity and 0.0t-1gini_d to 14.0t-1gini_d represent Diversity-ranked Word Diversity. The red (blue) colour represents where the topic score (benchmark) model outperformed the benchmark (topic score) model.

As discussed previously, we have developed four different topic score measures to understand whether the chance of topics occurring in the documents, the word composition in each topic, and the topic distributions in the documents contribute into the prediction of volatility. The graph shows no dominant topic scores along the predicted days. However, it should be noted that Topic Diversity is never chosen due to the lower *R*^2^ compared with other measures. This means that, compared to the other measures, topic diversity may not be an appropriate measure. The same conclusion of no dominant topic scores remains when we zoom out and check all the 1032 days with the public data. The results shown in Fig 14 indicates that there is no one prevailing type of topic score measure that is consistently better than the other types if the threshold is set as 0 as the chosen predictors in each prediction day as shown by the reds and the blues are all scattered.

To conclude, the result from the public data shows a few deficiencies: the few days that the topic score model is chosen over the benchmark, the inability to interpret with some critical events; and the inconsistent chosen topic score measures. However, we notice that using private data allows us to address some of these deficiencies because private data is considered to be more informative with less noise compared to the public data. Furthermore, as shown in Table 5, the result from the private data contains more observations when the topic score model is chosen over the benchmark model, enabling us to better investigate the usefulness of our proposed model. The result of the best model from the private data is from *model* 2 with a window size of 60 at threshold 0.45, shown in Table 5. We notice that the conditional RMSE ratio is lower than 1 but the conditional MAE ratio is higher than 1. However, we believe that the conditional MAE ratio is a more robust measure and can help to alleviate the impact from extreme volatility, according to [35]. In addition, we can see that in contrast to the 24-day observations from the public data, there are 67-day observations from the private data where topic score models were chosen over the benchmark model. Given that the total number of prediction days is 913 days in this shorter period in the licensed dataset, this indicates that the proportion of applicable days for prediction is higher: from only 2% of the predicted days made use of the topic score model from the public data and more than 7% of the prediction days adopted the topic score models in the private data.

**Fig 15:**
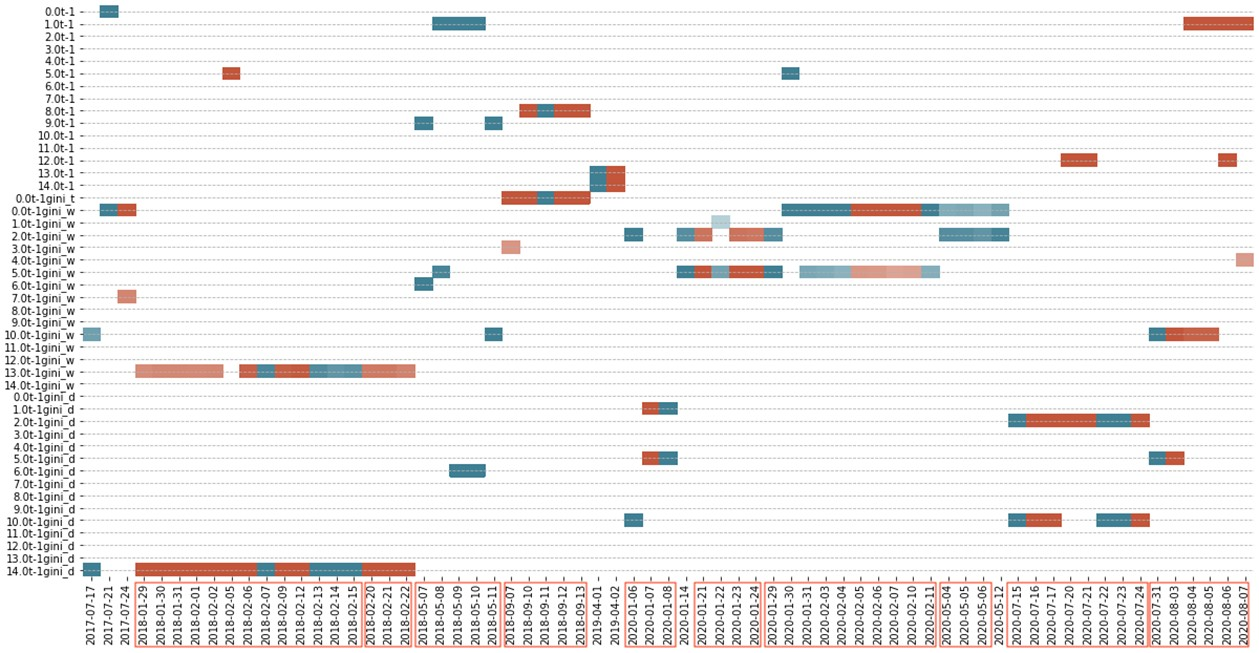
Different topic score chosen for prediction in the best model from the private data. The graph indicates the topic score measures were chosen for the predictions and the dates in the x-axis representing the last day of the regression period. The red (blue) colour represents where the topic score (benchmark) model outperformed the benchmark (topic score) model.

**Fig 16:**
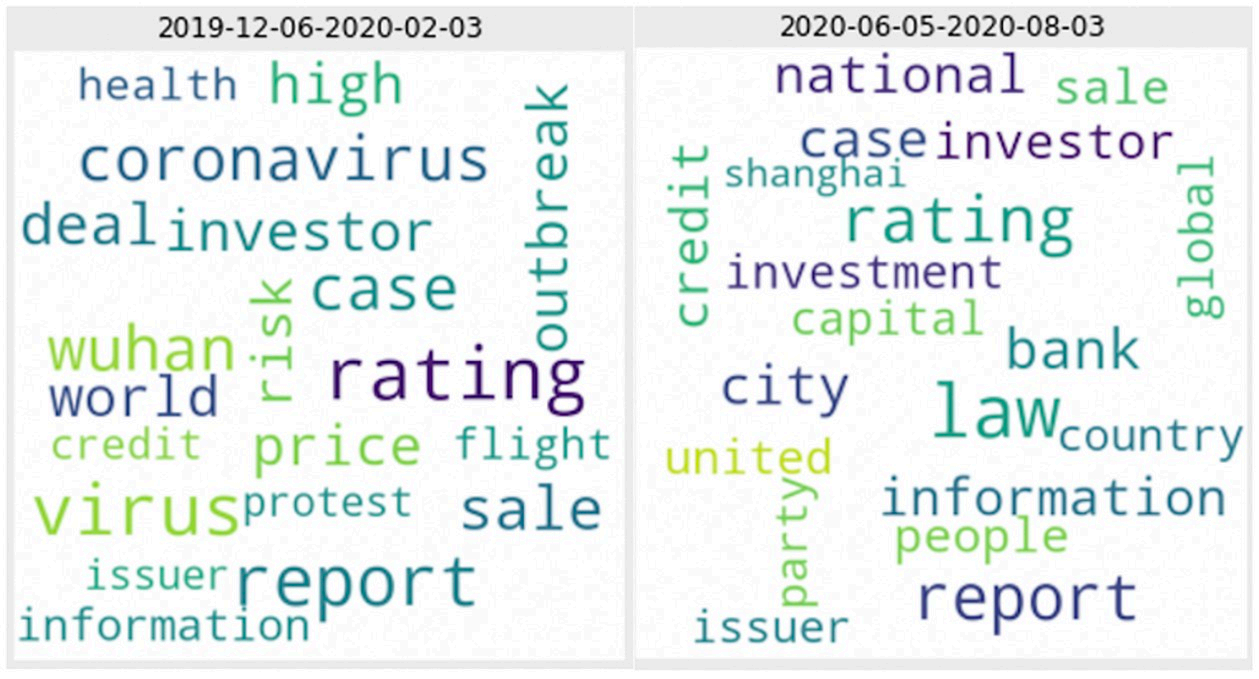
Word cloud from 2019-12-06 to 2020-02-03 and 2020-06-05 to 2020-08-03. These word clouds show the words from 2019-12-06 to 2020-02-03 and 2020-06-05 to 2020-08-03 in which their topic distributions and word distributions are used to constructed different topic score measures for the predictions on 2020-02-04 and 2020-08-04, respectively.

Fig 15 also shows that the prediction dates appear to cluster together and the 67-day observations from the private data can be mainly clustered into ten periods. By examining the topic words from the prediction periods, we notice that there may be some critical or large events that contributed to the prediction of the unexplained residuals from GARCH models. For example, from Fig 16 indicates that some of the words from the period 2019–12-06 to 2020–02-03 are “coronavirus” and “protest”. The appearance of these highly uncommon words during that period is potentially helpful in predicting the unexplained variance (i.e., the log of absolute standardized GARCH residuals) as the effect of these events might not be entirely reflected in the market price and volatility. Not only can we interpret some of the events in predicting volatility using the private data, but we also found that topic diversity is put into implementation in the topic score model for five predicted dates in autumn 2018. Four of the days even shows positive results, indicating that topic diversity helps to predict our volatility measure in the private dataset. However, the conclusion from the result of public data that no specific topic score measure dominates the prediction power still holds even when private data is used because the chosen topic score measures are also scattered in this graph.

**Fig 17:**
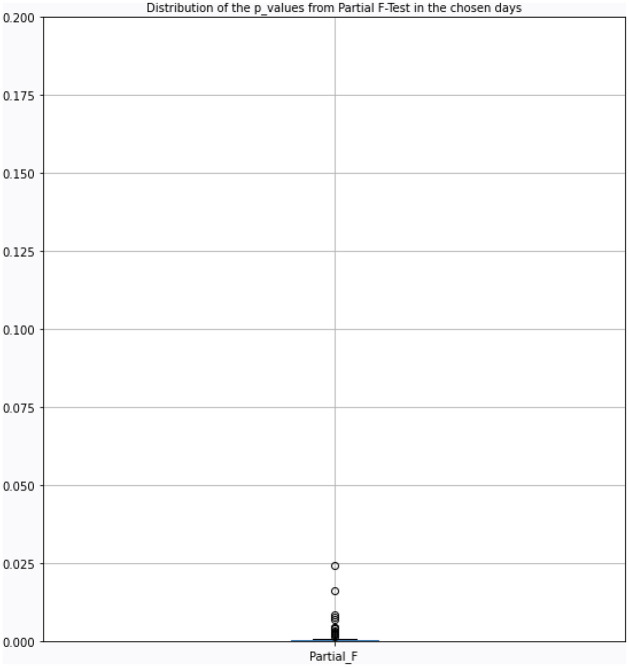
Boxplot of the p-values from the partial F-Test on the days where topic score models are chosen over the benchmark for the private data.

Given that there are over 30 observations when the topic score model is chosen, further statistical analysis is more meaningful in showing the usefulness of the topic score measures in addition to our discussed evaluation metrics. We have performed the partial F-Test on the days where the topic score model is used, to investigate whether the topic score model is significantly different from the benchmark model. On those predicted days, the restricted model of the partial F-Test is the benchmark model stated in Eq 6, while the full model is the topic score model stated in Eq 4. The result from the partial F test is shown in Fig 17, showing all the p-values are smaller than 0.05. Since a lower p-value means that the null hypothesis that the coefficients of the chosen features are 0 is rejected, it is obvious that the model with the best subset has most of the null hypothesis being rejected at the significant level of 5%. This means that best subset model is helpful in selecting useful predictors and therefore, in improving the performance of the predictions.

Observations of the performance of the public data and private data indicate that the private data give a better result in terms of a higher adoption rate of the topic score models and a better interpretation of the events during regression periods. This could be attributable to the better textual quality as the private data is single-sourced with more number of documents per day, while the public data is multiple-sourced with fewer documents.

## Discussion

In summary, we have introduced a machine learning pipeline that makes use of collected textual data to predict the market volatility using two rolling window models—a rolling LDA model and a rolling regression model. From our rolling LDA model, which is based on the LDA created by [15], we can extract the contextual information from the textual data in terms of latent topics from each window to predict the standardized GARCH residuals. The implementation of the rolling window scheme allows us to capture the dynamic behaviour and the changing context, of the documents by summarizing the probability of a certain topic’s occurrence for each document and the word distributions of each topic over the collection period. We also introduced four different types of topic scores measures—Popularity, Word Diversity, Topic Diversity, and Diversity-ranked Word Diversity—using the statistical output in an attempt to understand whether the diversity and popularity of words and topics matters in the Hong Kong stock market. By formulating and determining the topic scores measures from the statistical relationship between the documents and the topics, the need to manually describe the meaning of each topic can be eliminated, and thus the proposed methodology is a more flexible pipeline for using textual information to predict volatility.

In addition to the rolling LDA model, we also adopted a rolling regression model in order to capture the non-linearity of the stock market. Given the hypothesis that topic scores as predictors may not be helpful all the time, a hybrid model was adopted using several decision rules. If a signal shows that topic scores help, we adopt the model with topic scores; otherwise, the benchmark model, which is simply a regression model with the rolling mean of volatility as the predictor, is adopted. In the empirical study, we collected different types of news from both open sources on both the internet and licensed database as the textual data and used the HSI as the financial data to model the volatility measure by GARCH. The results show that the performance of the topic score models was occasionally better than that of the benchmark model. In other words, the news we collected can help to predict the volatility proxy, that is, the log of absolute standardized GARCH residuals of the HSI in specific periods. The findings of the results using the first dataset from open sources, the findings are not particularly strong—there is no clear evidence implying that the model with topic scores performs consistently better, as only 2% of the predicted days made use of the topic score model. However, we notice that better results are shown using the second dataset from the licensed database. Not only are there more observations showing that over 7% of the prediction days have adopted the topic score model, the words from the regression periods also allow better interpretation of the events happened and provide more clarity and insights into what might potentially be the driver of the volatility.

Our hybrid model approach yields better results and has stronger prediction power on more specific kinds of both textual and financial data, rather than the multiple-sourced, publicly available news that we used in the first dataset. As proven by the second dataset from a licensed database, the adoption rate for topic score models is higher and has better interpretation of events. We believe that by using private sources or specific sources and the portfolio returns or prices of single stocks, the noise of both the textual data and financial data can be mitigated and potentially reduced. For example, we could treat the return of a portfolio of real-estate stocks as the response (Y) and the corresponding real-estate news as the predictors (X). Given that the pipeline is flexible, it will be useful to try inputting data from various sectors or various portfolio combinations.

Another approach that may improve the prediction performance would be to enhance the topic scores selection process. In our empirical research, we only used up to “the best subset of two” to acquire a set of predictors, leading to limited prediction power. However, we suggest that it would be worthwhile to use either different variable selection algorithms or to increase the computational power of the best subset algorithm. For example, we could use forward or backward selection instead of best subset selection or to adopt a MapReduce technique to enable a comparison between more subsets, taking less time. This would allow the regression model to strike a better balance between under-fitting and over-fitting and should result in better performance.

In conclusion, using the proposed methodology, the textual data we collected helped to predict the market volatility generated from the HSI. Given the fact that licensed data can achieve a better result than open-source data, we believe the nature and quality of the textual data are of paramount importance to the prediction model. As a result, in the future, we hope to effect an improvement in the prediction ability by inputting more specific kinds of data and, if plausible, by changing the topic scores selection algorithm.

## Supporting information

S1 File (PDF)
