# Supplementary material for: Predicting standardized absolute returns using rolling-sample textual modelling
Source: PLoS One. 2021 Dec 7;16(12):e0260132. doi: 10.1371/journal.pone.0260132 (PMC8651148; doi:10.1371/journal.pone.0260132)
Supplement: S1 File — (PDF) [file pone.0260132.s001.pdf]

Supporting information

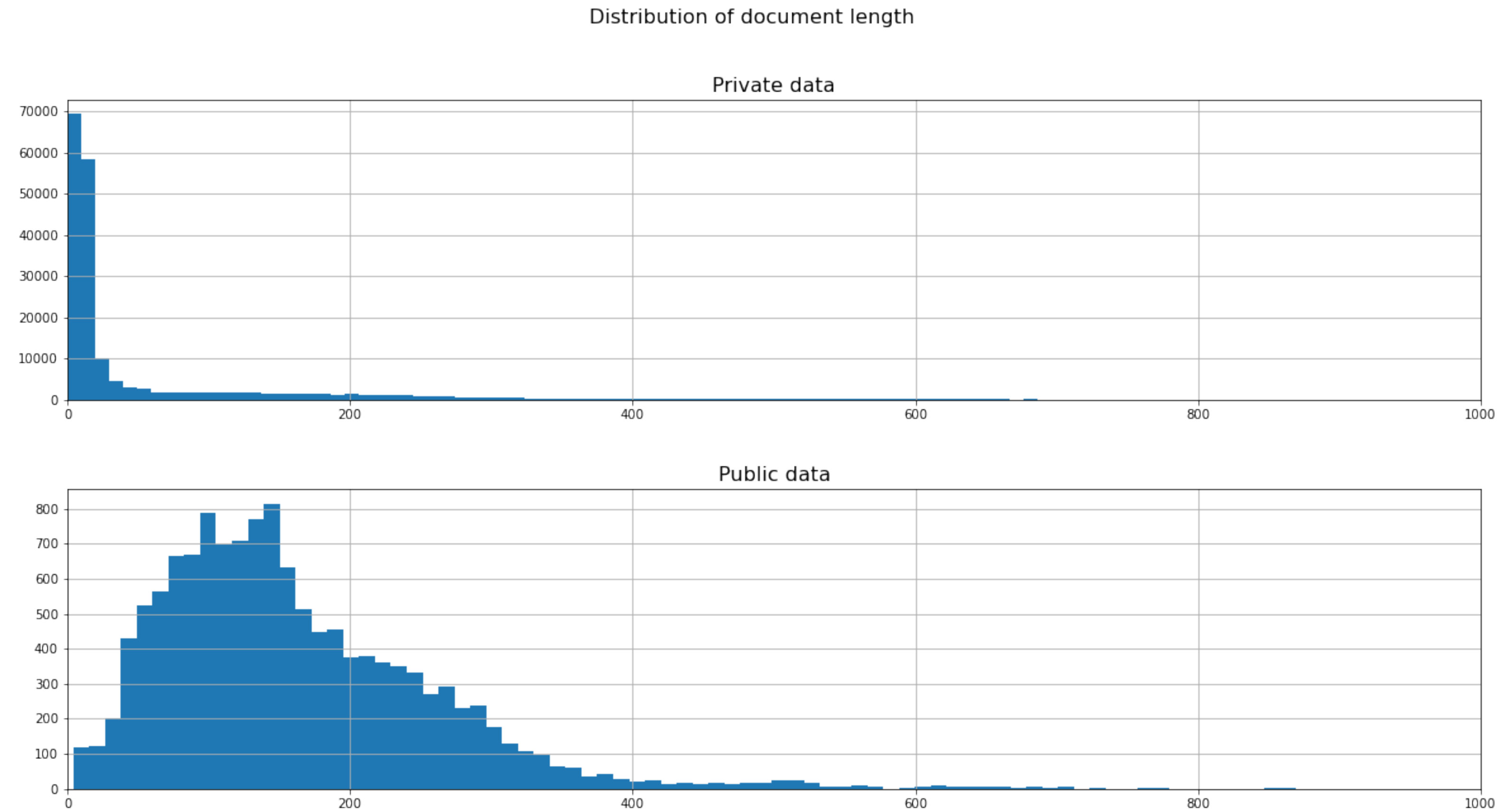

Fig 18. Distribution of the length of documents in the pivate data and public data respectively after the entire data cleansing procedure

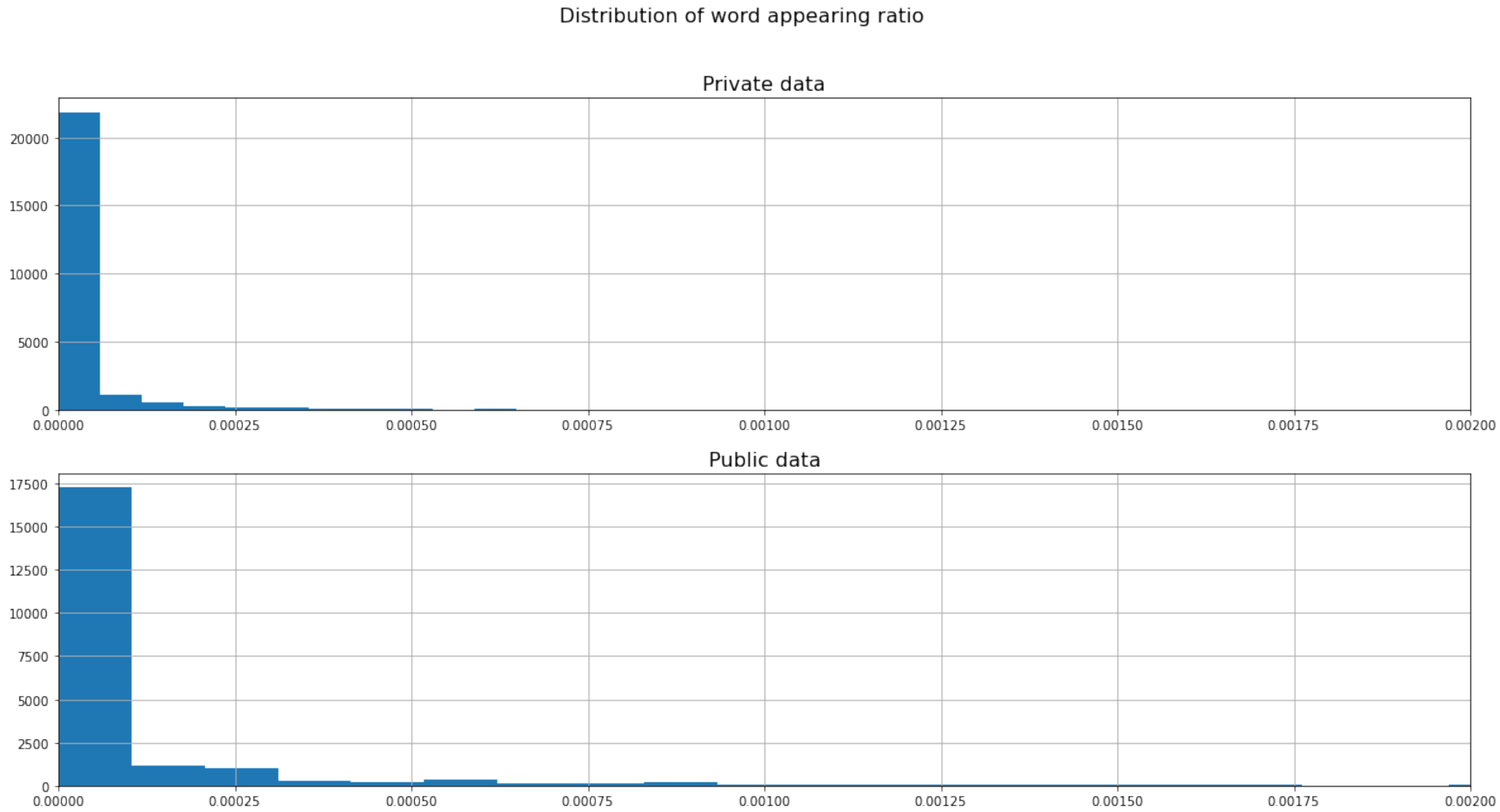

Fig 19. The word appearing ratio for the words in the private data and public data respectively. The ratio shows the uniqueness of each word by the following calculation: $word\ appearing\ ratio\ =\ number\ of\ times\ a\ document\ contains\ the\ word\ /\ number\ of\ documents\ in\ the\ dataset$

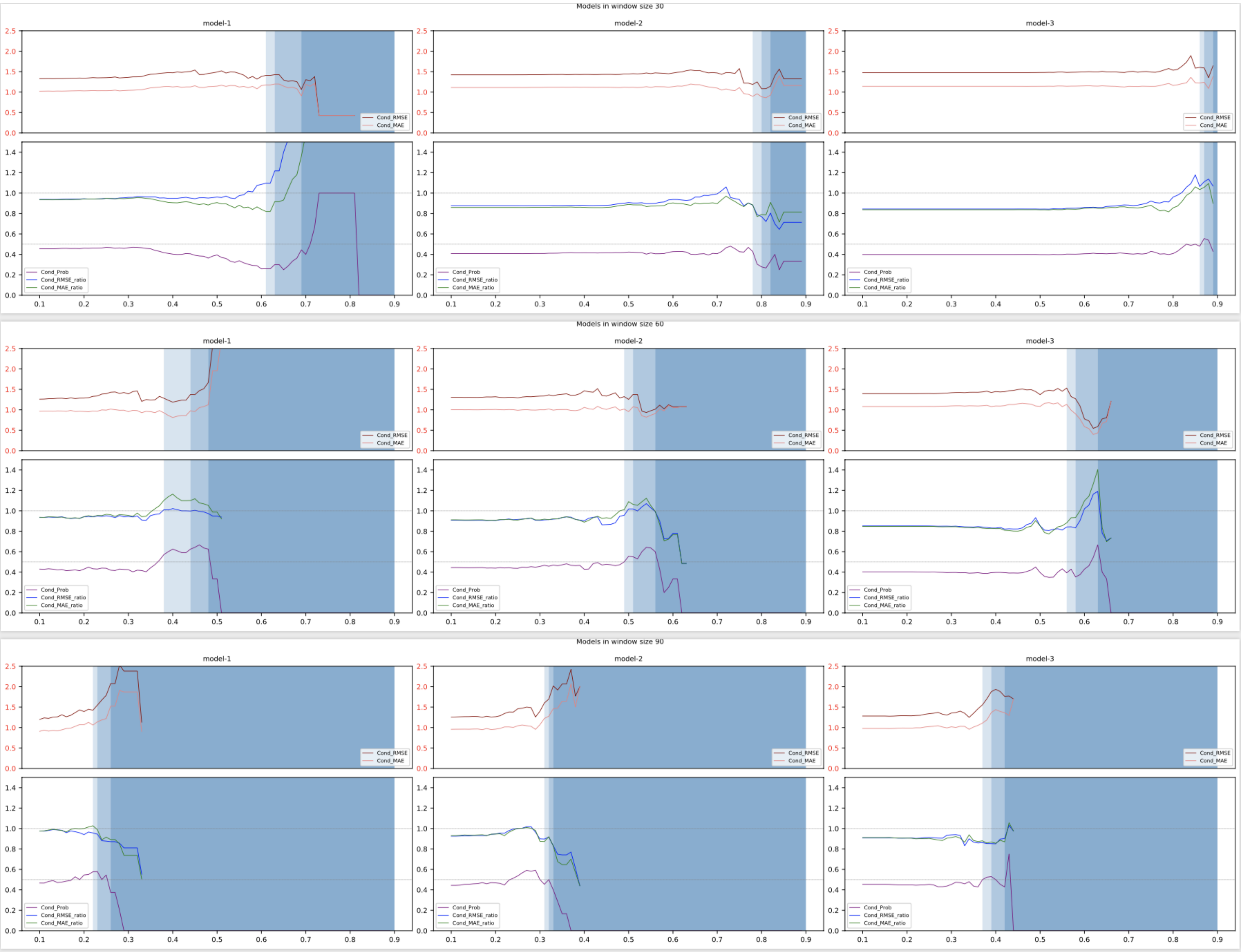

Fig 20. Model performance vs benchmark performance in the window sizes of 30, 60, and 90 days for public data

These graphs show the performance of the public data in the window sizes of 30, 60, and 90 days where in each graph, the y-axis indicates the results of the five evaluation measures and the x axis indicates the corresponding threshold. Under the graph of each model, the top graph shows the conditional RMSE and the conditional MAE, and the bottom graph shows the RMSE ratio, the MAE ratio, and the conditional probability. Tthe shaded area indicates the number of days that the topic model was chosen. The darkest blue, mid blue, the lightest blue and white areas represent 0-10, 10-20, 20-30 and above 30 observations, respectively.

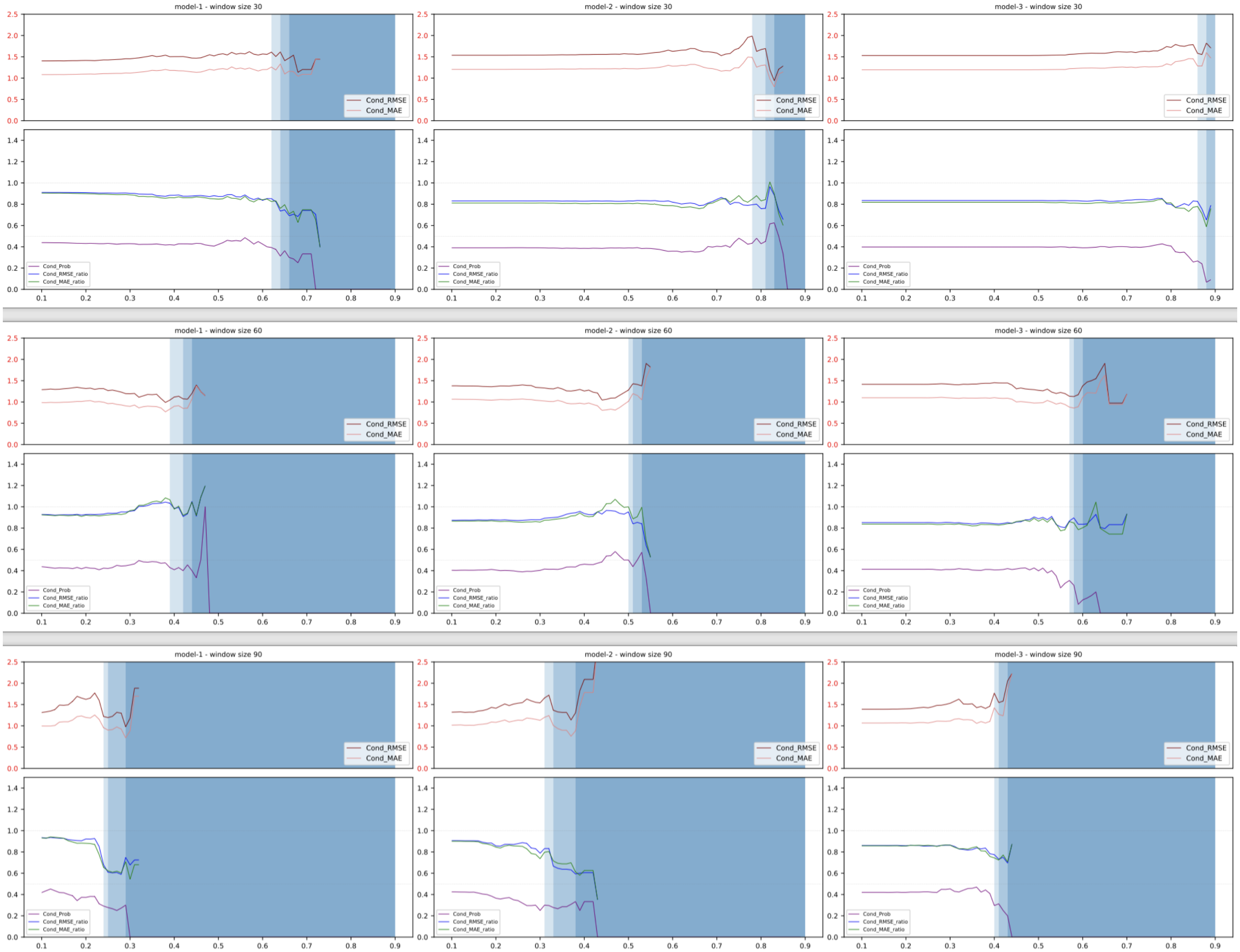

Fig 21. Model performance vs benchmark performance in the window sizes of 30, 60, and 90 days for private data

These graphs show the performance of the private data in the window sizes of 30, 60, and 90 days where in each graph, the y-axis indicates the results of the five evaluation measures and the x axis indicates the corresponding threshold. Under the graph of each model, the top graph shows the conditional RMSE and the conditional MAE, and the bottom graph shows the RMSE ratio, the MAE ratio, and the conditional probability. The shaded area indicates the number of days that the topic model was chosen. The darkest blue, mid blue, the lightest blue and white areas represent 0-10, 10-20, 20-30 and above 30 observations, respectively.
